# Supplementary material for: Genomic and clinical characteristics of campylobacteriosis in Australia
Source: Microb Genom. 2024 Jan 12;10(1):001174. doi: 10.1099/mgen.0.001174 (PMC10868609; doi:10.1099/mgen.0.001174)

Supporting Information

S1 Table. Summary of Campylobacter isolates (n = 1122) included in this study.

S2 Table: Antimicrobial resistance genes and mutations used to infer phenotypic resistance in Campylobacter isolates.

S3 Table. Univariable results for hospitalisation, adjusted for age group, sex, and location, and final multivariable model. OR: odds ratio, aOR: adjusted odds ratio, CI: confidence interval, ref: reference category, Inf: no limit on confidence interval.

S4 Table. Univariable results for prescription of antibiotics following illness, adjusted for age group, sex, and location, and final multivariable model. OR: odds ratio, aOR: adjusted odds ratio, CI: confidence interval, ref: reference category, Inf: no limit on confidence interval.

S5 Table. Univariable results for length of diarrhoeal illness, adjusted for age group, sex, and location, and final multivariable model. OR: odds ratio, aOR: adjusted odds ratio, CI: confidence interval, ref: reference category, Inf: no limit on confidence interval.

S6 Table: Summary of multi-locus sequence type (MLST) and virulence gene prevalence in *Campylobacter jejuni* and *C. coli* human isolates.

S7 Table. Comparison of isolate virulence gene prevalence between human and retail meat isolates, Australia, 2018–2019. N/A: no gene present, or no analysis possible due to zero levels. * indicates significant result ($p < 0.05$).

S1 File. Random Forest model outputs for *Campylobacter jejuni* and *C. coli* determining virulence genes that predict hospitalisation or length of diarrhoeal illness in Australia, 2018–2019.

S2 File. Random Forest model outputs determining virulence genes that predict a human case compared with retail meat and offal isolates in Australia, 2018–2019.

S1 Fig. Prevalence of virulence genes and gene clustering by multi-locus sequence type (MLST) for *C. jejuni* human isolates. The colour scale represents the proportion of isolates within each MLST expressing each virulence gene.

S2 Fig. Prevalence of virulence genes and gene clustering by multi-locus sequence type (MLST) for *C. coli* human isolates. The colour scale represents the proportion of isolates within each MLST expressing each virulence gene.

S3 Fig. Maximum likelihood phylogenetic tree showing the core genome relationship between *C. jejuni* isolates (*n* = 422) from humans in the Australian Capital Territory (ACT), New South Wales (NSW), and Queensland (Qld). The circle lanes from inner to outer indicate jurisdiction, multilocus sequence type (MLST), the number of class of antimicrobial genotype detected, and the number and trait class of virulence genotype detected.

S4 Fig. Maximum likelihood phylogenetic tree showing the core genome relationship between *C. coli* isolates (*n* = 84) from humans in the Australian Capital Territory (ACT), New South Wales (NSW), and Queensland (QLD). The circle lanes from inner to outer indicate jurisdiction, multilocus sequence type (MLST), the number and class of antimicrobial genotype detected, and the number and trait class of virulence genotype detected.

S5 Fig. Relative importance by Gini* coefficient of *Campylobacter jejuni* virulence genes for predicting case isolates^ compared with meat isolates in Australia, 2018–2019. * Mean decrease in Gini coefficient measures how much of each variable contributes to the homogeneity of the nodes and leaves in the random forest. The higher the value of mean decrease Gini score, the higher the importance of the variable. Values should be considered relative to those of other variables rather than absolute values. ^Genes more common in case isolates include fliK, Cj1136, Cj1138, Cj1135, maf4, neuC, rfbC, wlaN, cysC, Cj1422c, Cj1421c, gmhA2, kpsC, waaV, fcl, Cj1420c, ciaB, and Cj1419c although not all differences in gene prevalence are significant ($p < 0.05$).

Table S1. Summary of *Campylobacter* isolates (*n* = 1122) included in this study.

Isolate no.	Year of isolation	Source	Species	BioProject	Accession no.
18A1001H1	2018	Human	C. coli	PRJNA592186	SRR10542455
18A1002H1	2018	Human	C. jejuni	PRJNA592186	SRR10542454
18A1004H1	2018	Human	C. jejuni	PRJNA592186	SRR10542375
18A1005H1	2018	Human	C. jejuni	PRJNA592186	SRR10542264
18A1006H1	2018	Human	C. jejuni	PRJNA592186	SRR10542153
18A1007H1	2018	Human	C. jejuni	PRJNA592186	SRR10542042
18A1008H1	2018	Human	C. jejuni	PRJNA592186	SRR10541999
18A1009H1	2018	Human	C. coli	PRJNA592186	SRR10541988
18A1010H1	2018	Human	C. jejuni	PRJNA592186	SRR10541977
18A1011H1	2018	Human	C. jejuni	PRJNA592186	SRR10541966
18A1013H1	2018	Human	C. jejuni	PRJNA592186	SRR10542453
18A1014H1	2018	Human	C. coli	PRJNA592186	SRR10542442
18A1015H1	2018	Human	C. jejuni	PRJNA592186	SRR10542431
18A1016H1	2018	Human	C. jejuni	PRJNA592186	SRR10542484
18A1017H1	2018	Human	C. jejuni	PRJNA592186	SRR10542473
18A1018H1	2018	Human	C. jejuni	PRJNA592186	SRR10542462
18A1024H1	2018	Human	C. jejuni	PRJNA592186	SRR10542408
18A1025H1	2018	Human	C. jejuni	PRJNA592186	SRR10542397
18A1026H1	2018	Human	C. jejuni	PRJNA592186	SRR10542386
18A1029H1	2018	Human	C. jejuni	PRJNA592186	SRR10542374
18A1032H1	2018	Human	C. jejuni	PRJNA592186	SRR10542352
18A1033H1	2018	Human	C. jejuni	PRJNA592186	SRR10542341
18A1034H1	2018	Human	C. jejuni	PRJNA592186	SRR10542330
18A1035H1	2018	Human	C. jejuni	PRJNA592186	SRR10542319
18A1036H1	2018	Human	C. jejuni	PRJNA592186	SRR10542308
18A1037H1	2018	Human	C. jejuni	PRJNA560409	SRR9974819
18A1038H1	2018	Human	C. jejuni	PRJNA560409	SRR9974818
18A1039H1	2018	Human	C. jejuni	PRJNA560409	SRR9974811
18A1040H1	2018	Human	C. jejuni	PRJNA560409	SRR9974810
18A1041H1	2018	Human	C. jejuni	PRJNA560409	SRR9974802
18A1043H1	2018	Human	C. jejuni	PRJNA560409	SRR9974803
18A1044H1	2018	Human	C. jejuni	PRJNA560409	SRR9974800
18A1045H1	2018	Human	C. jejuni	PRJNA560409	SRR9974801
18A1046H1	2018	Human	C. coli	PRJNA560409	SRR9974817
18A1047H1	2018	Human	C. jejuni	PRJNA560409	SRR9974806
18A1048H1	2018	Human	C. coli	PRJNA560409	SRR9974816
18A1049H1	2018	Human	C. jejuni	PRJNA560409	SRR9974807
18A1050H1	2018	Human	C. jejuni	PRJNA560409	SRR9974804
18A1052H1	2018	Human	C. jejuni	PRJNA560409	SRR9974805
18A1053H1	2018	Human	C. jejuni	PRJNA560409	SRR9974794
18A1056H1	2018	Human	C. jejuni	PRJNA560409	SRR9974795
18A1057H1	2018	Human	C. jejuni	PRJNA560409	SRR9974786
18A1058H1	2018	Human	C. jejuni	PRJNA560409	SRR9974785
18N1001H1	2018	Human	C. coli	PRJNA592186	SRR10542030
18N1002H1	2018	Human	C. jejuni	PRJNA592186	SRR10542019
18N1003H1	2018	Human	C. coli	PRJNA592186	SRR10542008
18N1005H1	2018	Human	C. jejuni	PRJNA592186	SRR10542004
18N1007H1	2018	Human	C. jejuni	PRJNA592186	SRR10542002
18N1010H1	2018	Human	C. jejuni	PRJNA592186	SRR10541998
18N1011H1	2018	Human	C. jejuni	PRJNA592186	SRR10541997
18N1015H1	2018	Human	C. jejuni	PRJNA592186	SRR10541994
18N1017H1	2018	Human	C. jejuni	PRJNA592186	SRR10541993
18N1018H1	2018	Human	C. jejuni	PRJNA592186	SRR10541992
18N1019H1	2018	Human	C. jejuni	PRJNA592186	SRR10541991
18N1020H1	2018	Human	C. coli	PRJNA592186	SRR10541990
18N1021H1	2018	Human	C. jejuni	PRJNA592186	SRR10541989
18N1023H1	2018	Human	C. jejuni	PRJNA592186	SRR10541986
18N1029H1	2018	Human	C. jejuni	PRJNA592186	SRR10541982
18N1032H1	2018	Human	C. jejuni	PRJNA592186	SRR10541979
18N1033H1	2018	Human	C. coli	PRJNA592186	SRR10541978
18N1034H1	2018	Human	C. jejuni	PRJNA592186	SRR10541976
18N1039H1	2018	Human	C. jejuni	PRJNA592186	SRR10541973
18N1043H1	2018	Human	C. jejuni	PRJNA592186	SRR10541970
18N1045H1	2018	Human	C. coli	PRJNA592186	SRR10541969
18N1049H1	2018	Human	C. jejuni	PRJNA592186	SRR10541968
18N1050H1	2018	Human	C. jejuni	PRJNA592186	SRR10541967
18N1051H1	2018	Human	C. jejuni	PRJNA592186	SRR10541965
18N1053H1	2018	Human	C. coli	PRJNA592186	SRR10541964
18N1054H1	2018	Human	C. coli	PRJNA592186	SRR10541963
18N1055H1	2018	Human	C. coli	PRJNA592186	SRR10541962
18N1057H1	2018	Human	C. jejuni	PRJNA592186	SRR10541961
18N1062H1	2018	Human	C. jejuni	PRJNA592186	SRR10541960
18N1064H1	2018	Human	C. jejuni	PRJNA592186	SRR10541959
18N1065H1	2018	Human	C. jejuni	PRJNA592186	SRR10541958
18N1066H1	2018	Human	C. jejuni	PRJNA592186	SRR10541957
18N1070H1	2018	Human	C. jejuni	PRJNA592186	SRR10541956
18N1071H1	2018	Human	C. jejuni	PRJNA592186	SRR10542452
18N1072H1	2018	Human	C. jejuni	PRJNA592186	SRR10542451
18N1076H1	2018	Human	C. jejuni	PRJNA592186	SRR10542450
18N1078H1	2018	Human	C. jejuni	PRJNA592186	SRR10542449
18N1079H1	2018	Human	C. jejuni	PRJNA592186	SRR10542448
18N1080H1	2018	Human	C. jejuni	PRJNA592186	SRR10542447
18N1081H1	2018	Human	C. jejuni	PRJNA592186	SRR10542446
18N1082H1	2018	Human	C. jejuni	PRJNA592186	SRR10542445
18N1086H1	2018	Human	C. jejuni	PRJNA592186	SRR10542444
18N1087H1	2018	Human	C. jejuni	PRJNA592186	SRR10542443
18N1088H1	2018	Human	C. jejuni	PRJNA592186	SRR10542441
18N1089H1	2018	Human	C. coli	PRJNA592186	SRR10542440
18N1089H2	2018	Human	C. jejuni	PRJNA592186	SRR10542439
18N1091H1	2018	Human	C. coli	PRJNA592186	SRR10542438
18N1092H1	2018	Human	C. jejuni	PRJNA592186	SRR10542437
18N1093H1	2018	Human	C. jejuni	PRJNA592186	SRR10542436
18N1094H1	2018	Human	C. coli	PRJNA592186	SRR10542435
18N1097H1	2018	Human	C. jejuni	PRJNA592186	SRR10542434
18N1098H1	2018	Human	C. jejuni	PRJNA592186	SRR10542433
18N1099H1	2018	Human	C. coli	PRJNA592186	SRR10542432
18N1100H1	2018	Human	C. coli	PRJNA592186	SRR10542430
18N1101H1	2018	Human	C. jejuni	PRJNA592186	SRR10542429
18N1102H1	2018	Human	C. jejuni	PRJNA592186	SRR10542428

18N1103H1	2018	Human	C. jejuni	PRJNA592186	SRR10542427
18N1107H1	2018	Human	C. jejuni	PRJNA592186	SRR10542426
18N1113H1	2018	Human	C. jejuni	PRJNA560409	SRR9974743
18N1114H1	2018	Human	C. jejuni	PRJNA560409	SRR9974742
18N1115H1	2018	Human	C. jejuni	PRJNA560409	SRR9974749
18N1116H1	2018	Human	C. jejuni	PRJNA560409	SRR9974748
18N1119H1	2018	Human	C. jejuni	PRJNA592186	SRR10542425
18N1120H1	2018	Human	C. jejuni	PRJNA592186	SRR10542424
18N1121H1	2018	Human	C. jejuni	PRJNA592186	SRR10542487
18N1122H1	2018	Human	C. coli	PRJNA560409	SRR9974716
18N1125H1	2019	Human	C. jejuni	PRJNA560409	SRR9974713
18N1126H1	2019	Human	C. jejuni	PRJNA560409	SRR9974714
18N1128H1	2019	Human	C. jejuni	PRJNA560409	SRR9974712
18N1129H1	2019	Human	C. jejuni	PRJNA560409	SRR9974709
18N1133H1	2019	Human	C. jejuni	PRJNA560409	SRR9974694
18N1134H1	2019	Human	C. jejuni	PRJNA560409	SRR9974693
18N1135H1	2019	Human	C. jejuni	PRJNA560409	SRR9974692
18N1138H1	2019	Human	C. jejuni	PRJNA560409	SRR9974691
18Q1001H1	2018	Human	C. jejuni	PRJNA592186	SRR10542389
18Q1002H1	2018	Human	C. jejuni	PRJNA592186	SRR10542388
18Q1003H1	2018	Human	C. jejuni	PRJNA592186	SRR11318650
18Q1004H1	2018	Human	C. jejuni	PRJNA592186	SRR10542387
18Q1005H1	2018	Human	C. jejuni	PRJNA592186	SRR10542385
18Q1006H1	2018	Human	C. coli	PRJNA592186	SRR10542384
18Q1007H1	2018	Human	C. jejuni	PRJNA592186	SRR10542383
18Q1008H1	2018	Human	C. coli	PRJNA592186	SRR10542382
18Q1009H1	2018	Human	C. jejuni	PRJNA592186	SRR10542381
18Q1010H1	2018	Human	C. jejuni	PRJNA592186	SRR10542380
18Q1011H1	2018	Human	C. jejuni	PRJNA592186	SRR10542379
18Q1012H1	2018	Human	C. jejuni	PRJNA592186	SRR10542378
18Q1013H1	2018	Human	C. jejuni	PRJNA592186	SRR10542377
18Q1014H1	2018	Human	C. jejuni	PRJNA592186	SRR10542376
18Q1015H1	2018	Human	C. jejuni	PRJNA592186	SRR10542373
18Q1016H1	2018	Human	C. jejuni	PRJNA592186	SRR10542372
18Q1017H1	2018	Human	C. jejuni	PRJNA592186	SRR10542371
18Q1018H1	2018	Human	C. jejuni	PRJNA592186	SRR10542370
18Q1019H1	2018	Human	C. jejuni	PRJNA592186	SRR10542369
18Q1020H1	2018	Human	C. coli	PRJNA592186	SRR10542368
18Q1021H1	2018	Human	C. jejuni	PRJNA592186	SRR10542367
18Q1022H1	2018	Human	C. jejuni	PRJNA592186	SRR10542366
18Q1023H1	2018	Human	C. jejuni	PRJNA592186	SRR10542365
18Q1024H1	2018	Human	C. jejuni	PRJNA592186	SRR10542364
18Q1025H1	2018	Human	C. jejuni	PRJNA592186	SRR10542362
18Q1026H1	2018	Human	C. coli	PRJNA592186	SRR10542361
18Q1027H1	2018	Human	C. coli	PRJNA592186	SRR10542360
18Q1028H1	2018	Human	C. jejuni	PRJNA592186	SRR10542359
18Q1029H1	2018	Human	C. jejuni	PRJNA592186	SRR11318669
18Q1030H1	2018	Human	C. jejuni	PRJNA592186	SRR10542358
18Q1031H1	2018	Human	C. jejuni	PRJNA592186	SRR10542357
18Q1032H1	2018	Human	C. jejuni	PRJNA592186	SRR10542356
18Q1033H1	2018	Human	C. jejuni	PRJNA592186	SRR10542355
18Q1034H1	2018	Human	C. jejuni	PRJNA592186	SRR11318668
18Q1035H1	2018	Human	C. jejuni	PRJNA592186	SRR10542354
18Q1036H1	2018	Human	C. jejuni	PRJNA592186	SRR10542353
18Q1037H1	2018	Human	C. coli	PRJNA592186	SRR10542351
18Q1038H1	2018	Human	C. jejuni	PRJNA592186	SRR10542350
18Q1039H1	2018	Human	C. jejuni	PRJNA592186	SRR10542349
18Q1040H1	2018	Human	C. jejuni	PRJNA592186	SRR10542348
18Q1041H1	2018	Human	C. jejuni	PRJNA592186	SRR10542347
18Q1042H1	2018	Human	C. jejuni	PRJNA592186	SRR10542346
18Q1043H1	2018	Human	C. jejuni	PRJNA592186	SRR10542345
18Q1044H1	2018	Human	C. jejuni	PRJNA592186	SRR10542344
18Q1045H1	2018	Human	C. jejuni	PRJNA592186	SRR10542343
18Q1046H1	2018	Human	C. coli	PRJNA592186	SRR10542342
18Q1047H1	2018	Human	C. jejuni	PRJNA592186	SRR10542340
18Q1048H1	2018	Human	C. jejuni	PRJNA592186	SRR10542339
18Q1049H1	2018	Human	C. jejuni	PRJNA592186	SRR10542338
18Q1050H1	2018	Human	C. coli	PRJNA592186	SRR10542337
18Q1051H1	2018	Human	C. jejuni	PRJNA592186	SRR10542336
18Q1052H1	2018	Human	C. coli	PRJNA592186	SRR10542335
18Q1053H1	2018	Human	C. coli	PRJNA592186	SRR10542334
18Q1054H1	2018	Human	C. jejuni	PRJNA592186	SRR11318667
18Q1055H1	2018	Human	C. jejuni	PRJNA592186	SRR10542333
18Q1056H1	2018	Human	C. jejuni	PRJNA592186	SRR11318666
18Q1057H1	2018	Human	C. jejuni	PRJNA592186	SRR11318665
18Q1058H1	2018	Human	C. coli	PRJNA592186	SRR11318663
18Q1059H1	2018	Human	C. coli	PRJNA592186	SRR11318662
18Q1060H1	2018	Human	C. coli	PRJNA592186	SRR11318661
18Q1061H1	2018	Human	C. jejuni	PRJNA592186	SRR10542332
18Q1062H1	2018	Human	C. jejuni	PRJNA592186	SRR10542331
18Q1063H1	2018	Human	C. coli	PRJNA592186	SRR11318660
18Q1064H1	2018	Human	C. coli	PRJNA592186	SRR10542329
18Q1065H1	2018	Human	C. jejuni	PRJNA592186	SRR10542328
18Q1066H1	2018	Human	C. jejuni	PRJNA592186	SRR10542327
18Q1067H1	2018	Human	C. coli	PRJNA592186	SRR10542326
18Q1068H1	2018	Human	C. jejuni	PRJNA592186	SRR10542325
18Q1069H1	2018	Human	C. jejuni	PRJNA592186	SRR10542324
18Q1070H1	2018	Human	C. jejuni	PRJNA592186	SRR10542323
18Q1071H1	2018	Human	C. jejuni	PRJNA592186	SRR10542322
18Q1072H1	2018	Human	C. jejuni	PRJNA592186	SRR10542321
18Q1073H1	2018	Human	C. jejuni	PRJNA592186	SRR10542320
18Q1074H1	2018	Human	C. jejuni	PRJNA592186	SRR10542318
18Q1075H1	2018	Human	C. jejuni	PRJNA592186	SRR10542317
18Q1076H1	2018	Human	C. jejuni	PRJNA592186	SRR10542316
18Q1077H1	2018	Human	C. jejuni	PRJNA592186	SRR10542315
18Q1078H1	2018	Human	C. jejuni	PRJNA592186	SRR10542314
18Q1079H1	2018	Human	C. jejuni	PRJNA592186	SRR10542313
18Q1080H1	2018	Human	C. jejuni	PRJNA592186	SRR10542312
18Q1081H1	2018	Human	C. jejuni	PRJNA592186	SRR10542311
18Q1082H1	2018	Human	C. jejuni	PRJNA592186	SRR10542310
18Q1083H1	2018	Human	C. jejuni	PRJNA592186	SRR10542309
18Q1084H1	2018	Human	C. coli	PRJNA592186	SRR10542307

18Q1085H1	2018	Human	C. jejuni	PRJNA592186	SRR10542306
18Q1086H1	2018	Human	C. jejuni	PRJNA592186	SRR10542305
18Q1087H1	2018	Human	C. jejuni	PRJNA592186	SRR10542304
18Q1088H1	2018	Human	C. jejuni	PRJNA592186	SRR10542303
18Q1089H1	2018	Human	C. coli	PRJNA592186	SRR10542302
18Q1090H1	2018	Human	C. jejuni	PRJNA592186	SRR10542301
18Q1091H1	2018	Human	C. jejuni	PRJNA560409	SRR9974772
18Q1092H1	2018	Human	C. jejuni	PRJNA560409	SRR9974787
18Q1093H1	2018	Human	C. jejuni	PRJNA592186	SRR10542300
18Q1094H1	2018	Human	C. jejuni	PRJNA592186	SRR10542299
18Q1095H1	2018	Human	C. jejuni	PRJNA560409	SRR9974775
18Q1096H1	2018	Human	C. jejuni	PRJNA592186	SRR10542298
18Q1097H1	2018	Human	C. jejuni	PRJNA560409	SRR9974781
18Q1098H1	2018	Human	C. jejuni	PRJNA592186	SRR10542296
18Q1099H1	2018	Human	C. jejuni	PRJNA592186	SRR10542295
18Q1100H1	2018	Human	C. jejuni	PRJNA592186	SRR10542294
18Q1101H1	2018	Human	C. jejuni	PRJNA592186	SRR10542293
18Q1102H1	2018	Human	C. coli	PRJNA560409	SRR9974773
18Q1103H1	2018	Human	C. jejuni	PRJNA592186	SRR10542292
18Q1104H1	2018	Human	C. jejuni	PRJNA560409	SRR9974774
18Q1105H1	2018	Human	C. jejuni	PRJNA560409	SRR9974750
18Q1106H1	2018	Human	C. jejuni	PRJNA592186	SRR10542291
18Q1107H1	2018	Human	C. jejuni	PRJNA560409	SRR9974751
18Q1108H1	2018	Human	C. jejuni	PRJNA592186	SRR10542290
18Q1109H1	2018	Human	C. jejuni	PRJNA592186	SRR10542289
18Q1110H1	2018	Human	C. jejuni	PRJNA592186	SRR10542288
18Q1111H1	2018	Human	C. coli	PRJNA560409	SRR9974829
18Q1112H1	2018	Human	C. jejuni	PRJNA560409	SRR9974830
18Q1113H1	2018	Human	C. jejuni	PRJNA560409	SRR9974827
18Q1114H1	2018	Human	C. coli	PRJNA560409	SRR9974828
18Q1115H1	2018	Human	C. jejuni	PRJNA592186	SRR10542287
18Q1116H1	2018	Human	C. jejuni	PRJNA592186	SRR10542285
18Q1117H1	2018	Human	C. jejuni	PRJNA592186	SRR10542284
18Q1118H1	2018	Human	C. jejuni	PRJNA592186	SRR10542283
18Q1119H1	2018	Human	C. jejuni	PRJNA592186	SRR10542282
18Q1120H1	2018	Human	C. jejuni	PRJNA592186	SRR10542281
18Q1121H1	2018	Human	C. jejuni	PRJNA592186	SRR10542280
18Q1122H1	2018	Human	C. jejuni	PRJNA560409	SRR9974825
18Q1123H1	2018	Human	C. coli	PRJNA560409	SRR9974826
18Q1124H1	2018	Human	C. jejuni	PRJNA560409	SRR9974823
18Q1125H1	2018	Human	C. coli	PRJNA592186	SRR10542279
18Q1126H1	2018	Human	C. jejuni	PRJNA560409	SRR9974824
18Q1127H1	2018	Human	C. jejuni	PRJNA560409	SRR9974821
18Q1128H1	2018	Human	C. coli	PRJNA592186	SRR10542278
18Q1129H1	2018	Human	C. jejuni	PRJNA592186	SRR10542277
18Q1130H1	2018	Human	C. coli	PRJNA592186	SRR10542276
18Q1131H1	2018	Human	C. coli	PRJNA560409	SRR9974822
18Q1132H1	2018	Human	C. jejuni	PRJNA592186	SRR10542274
18Q1133H1	2018	Human	C. coli	PRJNA592186	SRR10542273
18Q1134H1	2018	Human	C. jejuni	PRJNA592186	SRR10542272
18Q1135H1	2018	Human	C. jejuni	PRJNA592186	SRR10542271

18Q1136H1	2018	Human	C. coli	PRJNA592186	SRR10542270
18Q1137H1	2018	Human	C. jejuni	PRJNA592186	SRR10542269
18Q1138H1	2018	Human	C. jejuni	PRJNA560409	SRR9974736
18Q1139H1	2018	Human	C. coli	PRJNA592186	SRR10542268
18Q1140H1	2018	Human	C. jejuni	PRJNA560409	SRR9974735
18Q1141H1	2018	Human	C. jejuni	PRJNA592186	SRR10542267
18Q1142H1	2018	Human	C. jejuni	PRJNA592186	SRR10542266
18Q1143H1	2018	Human	C. coli	PRJNA592186	SRR10542265
18Q1144H1	2018	Human	C. jejuni	PRJNA592186	SRR10542262
18Q1145H1	2018	Human	C. jejuni	PRJNA592186	SRR10542261
18Q1146H1	2018	Human	C. jejuni	PRJNA592186	SRR10542260
18Q1147H1	2018	Human	C. coli	PRJNA592186	SRR10542259
18Q1148H1	2018	Human	C. jejuni	PRJNA592186	SRR10542258
18Q1149H1	2018	Human	C. coli	PRJNA592186	SRR10542257
18Q1150H1	2018	Human	C. jejuni	PRJNA592186	SRR10542256
18Q1151H1	2018	Human	C. jejuni	PRJNA592186	SRR10542255
18Q1152H1	2018	Human	C. jejuni	PRJNA592186	SRR10542254
18Q1153H1	2018	Human	C. coli	PRJNA592186	SRR10542253
18Q1154H1	2018	Human	C. jejuni	PRJNA592186	SRR10542251
18Q1156H1	2018	Human	C. jejuni	PRJNA592186	SRR10542249
18Q1157H1	2018	Human	C. jejuni	PRJNA592186	SRR10542248
18Q1158H1	2018	Human	C. jejuni	PRJNA592186	SRR10542247
18Q1159H1	2018	Human	C. jejuni	PRJNA592186	SRR10542246
18Q1160H1	2018	Human	C. jejuni	PRJNA592186	SRR10542245
18Q1161H1	2018	Human	C. jejuni	PRJNA592186	SRR10542244
18Q1162H1	2018	Human	C. jejuni	PRJNA592186	SRR10542243
18Q1163H1	2018	Human	C. jejuni	PRJNA592186	SRR10542242
18Q1164H1	2018	Human	C. jejuni	PRJNA592186	SRR10542240
18Q1165H1	2018	Human	C. jejuni	PRJNA592186	SRR10542239
18Q1166H1	2018	Human	C. coli	PRJNA592186	SRR10542238
18Q1167H1	2018	Human	C. jejuni	PRJNA592186	SRR10542237
18Q1168H1	2018	Human	C. jejuni	PRJNA592186	SRR10542236
18Q1169H1	2018	Human	C. coli	PRJNA592186	SRR10542235
18Q1170H1	2018	Human	C. jejuni	PRJNA592186	SRR10542234
18Q1171H1	2018	Human	C. coli	PRJNA592186	SRR10542233
18Q1172H1	2018	Human	C. coli	PRJNA592186	SRR10542232
18Q1173H1	2018	Human	C. coli	PRJNA592186	SRR10542231
18Q1175H1	2018	Human	C. jejuni	PRJNA592186	SRR10542229
18Q1176H1	2018	Human	C. jejuni	PRJNA592186	SRR10542228
18Q1177H1	2018	Human	C. jejuni	PRJNA592186	SRR10542227
18Q1178H1	2018	Human	C. coli	PRJNA592186	SRR10542226
18Q1179H1	2018	Human	C. coli	PRJNA592186	SRR10542225
18Q1180H1	2018	Human	C. jejuni	PRJNA592186	SRR10542224
18Q1181H1	2018	Human	C. jejuni	PRJNA592186	SRR10542223
18Q1182H1	2018	Human	C. jejuni	PRJNA592186	SRR10542222
18Q1183H1	2018	Human	C. jejuni	PRJNA592186	SRR10542221
18Q1184H1	2018	Human	C. coli	PRJNA592186	SRR10542220
18Q1185H1	2018	Human	C. coli	PRJNA592186	SRR10542218
18Q1186H1	2018	Human	C. jejuni	PRJNA592186	SRR10542217
18Q1187H1	2018	Human	C. jejuni	PRJNA592186	SRR10542216
18Q1188H1	2018	Human	C. jejuni	PRJNA592186	SRR10542215

18Q1189H1	2018	Human	C. jejuni	PRJNA592186	SRR10542214
18Q1190H1	2018	Human	C. jejuni	PRJNA592186	SRR10542213
18Q1191H1	2018	Human	C. coli	PRJNA592186	SRR10542212
18Q1192H1	2018	Human	C. jejuni	PRJNA592186	SRR10542211
18Q1193H1	2018	Human	C. jejuni	PRJNA592186	SRR10542210
18Q1194H1	2018	Human	C. jejuni	PRJNA592186	SRR10542209
18Q1195H1	2018	Human	C. jejuni	PRJNA592186	SRR10542207
18Q1196H1	2018	Human	C. jejuni	PRJNA592186	SRR10542206
18Q1197H1	2018	Human	C. jejuni	PRJNA592186	SRR10542205
18Q1198H1	2018	Human	C. jejuni	PRJNA592186	SRR10542204
18Q1199H1	2018	Human	C. coli	PRJNA592186	SRR10542203
18Q1200H1	2018	Human	C. jejuni	PRJNA592186	SRR10542202
18Q1201H1	2018	Human	C. jejuni	PRJNA592186	SRR10542201
18Q1202H1	2018	Human	C. jejuni	PRJNA592186	SRR10542200
18Q1203H1	2018	Human	C. jejuni	PRJNA592186	SRR10542199
18Q1204H1	2018	Human	C. jejuni	PRJNA592186	SRR10542198
18Q1205H1	2018	Human	C. jejuni	PRJNA592186	SRR10542196
18Q1206H1	2018	Human	C. jejuni	PRJNA592186	SRR10542195
18Q1207H1	2018	Human	C. jejuni	PRJNA592186	SRR10542194
18Q1208H1	2018	Human	C. jejuni	PRJNA592186	SRR10542193
18Q1209H1	2018	Human	C. jejuni	PRJNA592186	SRR10542192
18Q1210H1	2018	Human	C. jejuni	PRJNA592186	SRR10542191
18Q1211H1	2018	Human	C. jejuni	PRJNA592186	SRR10542190
18Q1212H1	2018	Human	C. jejuni	PRJNA592186	SRR10542189
18Q1213H1	2018	Human	C. jejuni	PRJNA592186	SRR10542188
18Q1214H1	2018	Human	C. jejuni	PRJNA592186	SRR10542187
18Q1215H1	2018	Human	C. jejuni	PRJNA592186	SRR10542185
18Q1216H1	2018	Human	C. jejuni	PRJNA592186	SRR10542184
18Q1217H1	2018	Human	C. jejuni	PRJNA592186	SRR10542183
18Q1218H1	2018	Human	C. coli	PRJNA592186	SRR10542182
18Q1219H1	2018	Human	C. jejuni	PRJNA592186	SRR10542181
18Q1220H1	2018	Human	C. jejuni	PRJNA592186	SRR10542180
18Q1221H1	2018	Human	C. coli	PRJNA592186	SRR10542179
18Q1223H1	2018	Human	C. jejuni	PRJNA592186	SRR10542178
18Q1224H1	2018	Human	C. jejuni	PRJNA592186	SRR10542177
18Q1225H1	2018	Human	C. jejuni	PRJNA592186	SRR10542176
18Q1226H1	2018	Human	C. coli	PRJNA592186	SRR10542174
18Q1227H1	2018	Human	C. jejuni	PRJNA592186	SRR10542173
18Q1228H1	2018	Human	C. jejuni	PRJNA592186	SRR10542172
18Q1229H1	2018	Human	C. jejuni	PRJNA592186	SRR10542171
18Q1230H1	2018	Human	C. jejuni	PRJNA592186	SRR10542170
18Q1231H1	2018	Human	C. jejuni	PRJNA592186	SRR10542169
18Q1232H1	2018	Human	C. jejuni	PRJNA592186	SRR10542168
18Q1233H1	2018	Human	C. jejuni	PRJNA592186	SRR10542167
18Q1234H1	2018	Human	C. jejuni	PRJNA592186	SRR10542166
18Q1235H1	2018	Human	C. jejuni	PRJNA592186	SRR10542165
18Q1236H1	2018	Human	C. jejuni	PRJNA592186	SRR10542163
18Q1237H1	2018	Human	C. jejuni	PRJNA592186	SRR10542162
18Q1238H1	2018	Human	C. jejuni	PRJNA592186	SRR10542161
18Q1239H1	2018	Human	C. jejuni	PRJNA592186	SRR10542160
18Q1240H1	2018	Human	C. coli	PRJNA592186	SRR10542159
18Q1241H1	2018	Human	C. jejuni	PRJNA592186	SRR10542158
18Q1242H1	2018	Human	C. jejuni	PRJNA592186	SRR10542157
18Q1243H1	2018	Human	C. jejuni	PRJNA592186	SRR10542156
18Q1244H1	2018	Human	C. jejuni	PRJNA592186	SRR10542155
18Q1245H1	2018	Human	C. jejuni	PRJNA592186	SRR10542154
18Q1246H1	2018	Human	C. jejuni	PRJNA592186	SRR10542151
18Q1247H1	2018	Human	C. jejuni	PRJNA592186	SRR10542150
18Q1248H1	2018	Human	C. jejuni	PRJNA592186	SRR10542149
18Q1249H1	2018	Human	C. jejuni	PRJNA592186	SRR10542148
18Q1250H1	2018	Human	C. jejuni	PRJNA592186	SRR10542147
18Q1251H1	2018	Human	C. jejuni	PRJNA592186	SRR10542146
18Q1252H1	2018	Human	C. jejuni	PRJNA592186	SRR10542145
18Q1253H1	2018	Human	C. jejuni	PRJNA592186	SRR10542144
18Q1254H1	2018	Human	C. jejuni	PRJNA592186	SRR10542143
18Q1255H1	2018	Human	C. jejuni	PRJNA592186	SRR10542142
18Q1256H1	2018	Human	C. jejuni	PRJNA592186	SRR10542140
18Q1257H1	2018	Human	C. jejuni	PRJNA592186	SRR10542139
18Q1258H1	2018	Human	C. coli	PRJNA592186	SRR10542138
18Q1259H1	2018	Human	C. jejuni	PRJNA592186	SRR10542137
18Q1261H1	2018	Human	C. jejuni	PRJNA592186	SRR10542136
18Q1262H1	2018	Human	C. jejuni	PRJNA592186	SRR10542135
18Q1263H1	2018	Human	C. jejuni	PRJNA592186	SRR10542134
18Q1264H1	2018	Human	C. jejuni	PRJNA592186	SRR10542133
18Q1265H1	2018	Human	C. coli	PRJNA592186	SRR10542132
18Q1266H1	2018	Human	C. jejuni	PRJNA592186	SRR10542131
18Q1267H1	2018	Human	C. jejuni	PRJNA592186	SRR10542129
18Q1268H1	2018	Human	C. jejuni	PRJNA592186	SRR10542128
18Q1269H1	2018	Human	C. jejuni	PRJNA592186	SRR10542127
18Q1270H1	2018	Human	C. jejuni	PRJNA592186	SRR10542126
18Q1271H1	2018	Human	C. jejuni	PRJNA592186	SRR10542125
18Q1272H1	2018	Human	C. jejuni	PRJNA592186	SRR10542124
18Q1273H1	2018	Human	C. jejuni	PRJNA592186	SRR10542123
18Q1274H1	2018	Human	C. jejuni	PRJNA592186	SRR10542122
18Q1276H1	2018	Human	C. jejuni	PRJNA592186	SRR10542120
18Q1277H1	2018	Human	C. jejuni	PRJNA592186	SRR10542118
18Q1278H1	2018	Human	C. jejuni	PRJNA592186	SRR10542117
18Q1280H1	2018	Human	C. jejuni	PRJNA592186	SRR11318664
18Q1281H1	2018	Human	C. jejuni	PRJNA592186	SRR10542116
18Q1282H1	2018	Human	C. jejuni	PRJNA592186	SRR10542115
18Q1283H1	2018	Human	C. jejuni	PRJNA592186	SRR10542114
18Q1284H1	2018	Human	C. jejuni	PRJNA592186	SRR10542113
18Q1285H1	2018	Human	C. jejuni	PRJNA592186	SRR10542112
18Q1286H1	2018	Human	C. jejuni	PRJNA592186	SRR10542111
18Q1287H1	2018	Human	C. jejuni	PRJNA592186	SRR10542110
18Q1288H1	2018	Human	C. jejuni	PRJNA592186	SRR10542109
18Q1289H1	2018	Human	C. jejuni	PRJNA592186	SRR10542107
18Q1290H1	2018	Human	C. jejuni	PRJNA592186	SRR10542106
18Q1291H1	2018	Human	C. jejuni	PRJNA592186	SRR10542105
18Q1292H1	2018	Human	C. jejuni	PRJNA592186	SRR10542104
18Q1293H1	2018	Human	C. jejuni	PRJNA592186	SRR10542103
18Q1294H1	2018	Human	C. jejuni	PRJNA592186	SRR10542102

18Q1295H1	2018	Human	C. jejuni	PRJNA592186	SRR10542101
18Q1296H1	2018	Human	C. jejuni	PRJNA592186	SRR10542100
18Q1297H1	2018	Human	C. coli	PRJNA592186	SRR10542099
18Q1298H1	2018	Human	C. coli	PRJNA592186	SRR10542098
18Q1299H1	2018	Human	C. jejuni	PRJNA592186	SRR10542096
18Q1300H1	2018	Human	C. jejuni	PRJNA592186	SRR10542095
19A1001H1	2018	Human	C. jejuni	PRJNA560409	SRR9974784
19A1002H1	2018	Human	C. jejuni	PRJNA560409	SRR9974783
19A1006H1	2019	Human	C. jejuni	PRJNA592186	SRR10542297
19A1007H1	2019	Human	C. jejuni	PRJNA592186	SRR10542286
19A1008H1	2019	Human	C. jejuni	PRJNA592186	SRR10542275
19A1009H1	2019	Human	C. jejuni	PRJNA592186	SRR10542263
19A1012H1	2019	Human	C. jejuni	PRJNA592186	SRR10542252
19A1013H1	2019	Human	C. jejuni	PRJNA592186	SRR10542241
19A1014H1	2019	Human	C. jejuni	PRJNA592186	SRR10542230
19A1015H1	2019	Human	C. jejuni	PRJNA592186	SRR10542219
19A1016H1	2019	Human	C. jejuni	PRJNA592186	SRR10542208
19A1017H1	2019	Human	C. jejuni	PRJNA592186	SRR10542197
19A1018H1	2019	Human	C. jejuni	PRJNA592186	SRR10542186
19A1019H1	2019	Human	C. jejuni	PRJNA592186	SRR10542175
19A1020H1	2019	Human	C. jejuni	PRJNA592186	SRR10542164
19A1021H1	2019	Human	C. jejuni	PRJNA592186	SRR10542152
19A1022H1	2019	Human	C. jejuni	PRJNA592186	SRR10542141
19A1023H1	2019	Human	C. jejuni	PRJNA592186	SRR10542130
19A1024H1	2019	Human	C. jejuni	PRJNA592186	SRR10542119
19A1025H1	2019	Human	C. jejuni	PRJNA592186	SRR10542108
19A1026H1	2019	Human	C. jejuni	PRJNA592186	SRR10542097
19A1027H1	2019	Human	C. jejuni	PRJNA592186	SRR10542086
19A1028H1	2019	Human	C. jejuni	PRJNA592186	SRR10542075
19A1029H1	2019	Human	C. jejuni	PRJNA592186	SRR10542064
19A1030H1	2019	Human	C. jejuni	PRJNA592186	SRR10542053
19A1031H1	2019	Human	C. jejuni	PRJNA592186	SRR10542041
19N1002H1	2019	Human	C. jejuni	PRJNA592186	SRR10542486
19N1004H1	2019	Human	C. jejuni	PRJNA592186	SRR10542485
19N1005H1	2019	Human	C. jejuni	PRJNA592186	SRR10542483
19N1007H1	2019	Human	C. coli	PRJNA592186	SRR10542482
19N1008H1	2019	Human	C. jejuni	PRJNA592186	SRR10542481
19N1016H1	2019	Human	C. jejuni	PRJNA592186	SRR10542480
19N1025H1	2019	Human	C. coli	PRJNA592186	SRR10542479
19N1026H1	2019	Human	C. jejuni	PRJNA592186	SRR10542478
19N1027H1	2019	Human	C. jejuni	PRJNA592186	SRR10542477
19N1028H1	2019	Human	C. jejuni	PRJNA592186	SRR10542476
19N1029H1	2019	Human	C. jejuni	PRJNA592186	SRR10542475
19N1035H1	2019	Human	C. jejuni	PRJNA592186	SRR10542474
19N1036H1	2019	Human	C. jejuni	PRJNA592186	SRR10542472
19N1038H1	2019	Human	C. jejuni	PRJNA592186	SRR10542471
19N1039H1	2019	Human	C. jejuni	PRJNA592186	SRR10542470
19N1040H1	2019	Human	C. jejuni	PRJNA592186	SRR10542469
19N1042H1	2019	Human	C. jejuni	PRJNA592186	SRR10542468
19N1044H1	2019	Human	C. jejuni	PRJNA592186	SRR10542467
19N1048H1	2019	Human	C. jejuni	PRJNA592186	SRR10542466
19N1050H1	2019	Human	C. jejuni	PRJNA592186	SRR10542465
19N1055H1	2019	Human	C. jejuni	PRJNA592186	SRR10542464
19N1056H1	2019	Human	C. jejuni	PRJNA592186	SRR10542463
19N1063H1	2019	Human	C. jejuni	PRJNA592186	SRR10542460
19N1065H1	2019	Human	C. coli	PRJNA592186	SRR10542459
19N1069H1	2019	Human	C. jejuni	PRJNA592186	SRR10542458
19N1071H1	2019	Human	C. jejuni	PRJNA592186	SRR10542457
19N1072H1	2019	Human	C. jejuni	PRJNA592186	SRR11318670
19N1073H1	2019	Human	C. coli	PRJNA592186	SRR10542456
19N1074H1	2019	Human	C. jejuni	PRJNA592186	SRR10542423
19N1075H1	2019	Human	C. jejuni	PRJNA592186	SRR10542422
19N1076H1	2019	Human	C. jejuni	PRJNA592186	SRR10542421
19N1077H1	2019	Human	C. coli	PRJNA592186	SRR10542420
19N1078H1	2019	Human	C. coli	PRJNA592186	SRR10542418
19N1079H1	2019	Human	C. coli	PRJNA592186	SRR10542417
19N1080H1	2019	Human	C. coli	PRJNA592186	SRR11318658
19N1081H1	2019	Human	C. jejuni	PRJNA592186	SRR10542416
19N1082H1	2019	Human	C. jejuni	PRJNA592186	SRR10542415
19N1083H1	2019	Human	C. jejuni	PRJNA592186	SRR10542414
19N1084H1	2019	Human	C. jejuni	PRJNA592186	SRR10542413
19N1085H1	2019	Human	C. jejuni	PRJNA592186	SRR10542412
19N1087H1	2019	Human	C. jejuni	PRJNA592186	SRR10542411
19N1088H1	2019	Human	C. coli	PRJNA592186	SRR10542410
19N1090H1	2019	Human	C. jejuni	PRJNA592186	SRR10542409
19N1091H1	2019	Human	C. coli	PRJNA592186	SRR10542407
19N1093H1	2019	Human	C. coli	PRJNA592186	SRR10542406
19N1094H1	2019	Human	C. jejuni	PRJNA592186	SRR10542405
19N1096H1	2019	Human	C. jejuni	PRJNA592186	SRR10542404
19N1098H1	2019	Human	C. jejuni	PRJNA592186	SRR10542403
19N1099H1	2019	Human	C. jejuni	PRJNA592186	SRR10542402
19N1100H1	2019	Human	C. jejuni	PRJNA592186	SRR11318659
19N1101H1	2019	Human	C. jejuni	PRJNA592186	SRR10542401
19N1102H1	2019	Human	C. jejuni	PRJNA592186	SRR10542400
19N1103H1	2019	Human	C. jejuni	PRJNA592186	SRR10542399
19N1104H1	2019	Human	C. jejuni	PRJNA592186	SRR10542398
19N1107H1	2019	Human	C. coli	PRJNA592186	SRR10542396
19N1109H1	2019	Human	C. jejuni	PRJNA592186	SRR10542395
19N1111H1	2019	Human	C. jejuni	PRJNA592186	SRR10542394
19N1112H1	2019	Human	C. jejuni	PRJNA592186	SRR10542393
19N1113H1	2019	Human	C. jejuni	PRJNA592186	SRR10542392
19N1114H1	2019	Human	C. jejuni	PRJNA592186	SRR10542391
19N1115H1	2019	Human	C. jejuni	PRJNA592186	SRR10542390
19N1131H1	2019	Human	C. jejuni	PRJNA592186	SRR11318671
19N1186H1	2019	Human	C. jejuni	PRJNA592186	SRR11318656
19N1187H1	2019	Human	C. jejuni	PRJNA592186	SRR11318655
19N1188H1	2019	Human	C. jejuni	PRJNA592186	SRR11318654
19N1190H1	2019	Human	C. jejuni	PRJNA592186	SRR11318653
19N1191H1	2019	Human	C. coli	PRJNA592186	SRR11318657
19N1192H1	2019	Human	C. jejuni	PRJNA592186	SRR11318652
19N1193H1	2019	Human	C. jejuni	PRJNA592186	SRR11318651
19N3108F1	2019	Beef	C. coli	PRJNA591966	SRR10537610

17N3091F1	2017	Beef	C. coli	PRJNA591966	SRR10537765
17N3141F1	2017	Beef	C. coli	PRJNA591966	SRR10537755
17Q3003F1	2017	Beef	C. coli	PRJNA591966	SRR10537586
17N3026F1	2017	Chicken	C. coli	PRJNA591966	SRR10537805
17N3028F1	2017	Chicken	C. coli	PRJNA591966	SRR10537803
17Q3063F1	2017	Chicken	C. coli	PRJNA591966	SRR10537521
17Q3085F2	2017	Chicken	C. coli	PRJNA591966	SRR10537494
17V3001F1	2017	Chicken	C. coli	PRJNA591966	SRR10537995
17V3004F1	2017	Chicken	C. coli	PRJNA591966	SRR10537993
17V3005F1	2017	Chicken	C. coli	PRJNA591966	SRR10537992
17V3010F1	2017	Chicken	C. coli	PRJNA591966	SRR10537987
17V3016F1	2017	Chicken	C. coli	PRJNA591966	SRR10537979
17V3017F1	2017	Chicken	C. coli	PRJNA591966	SRR10537978
17V3024F1	2017	Chicken	C. coli	PRJNA591966	SRR10537970
17V3034F1	2017	Chicken	C. coli	PRJNA591966	SRR10537960
17V3036F1	2017	Chicken	C. coli	PRJNA591966	SRR10537958
17V3037F1	2017	Chicken	C. coli	PRJNA591966	SRR10537957
17V3038F1	2017	Chicken	C. coli	PRJNA591966	SRR10537956
17V3040F1	2017	Chicken	C. coli	PRJNA591966	SRR10537954
17V3041F1	2017	Chicken	C. coli	PRJNA591966	SRR10537953
17V3044F1	2017	Chicken	C. coli	PRJNA591966	SRR10537950
17V3046F1	2017	Chicken	C. coli	PRJNA591966	SRR10537949
17V3047F1	2017	Chicken	C. coli	PRJNA591966	SRR10537948
17V3049F1	2017	Chicken	C. coli	PRJNA591966	SRR10537946
17V3051F1	2017	Chicken	C. coli	PRJNA591966	SRR10537945
17V3056F1	2017	Chicken	C. coli	PRJNA591966	SRR10537941
17V3060F2	2017	Chicken	C. coli	PRJNA591966	SRR10537934
18N3092F1	2018	Chicken	C. coli	PRJNA591966	SRR10537640
18Q3016F1	2018	Chicken	C. coli	PRJNA591966	SRR10538031
18Q3017F1	2018	Chicken	C. coli	PRJNA591966	SRR10538030
18V3002F1	2018	Chicken	C. coli	PRJNA591966	SRR10537932
18V3009F1	2018	Chicken	C. coli	PRJNA591966	SRR10537925
18V3015F1	2018	Chicken	C. coli	PRJNA591966	SRR10537920
18V3033F1	2018	Chicken	C. coli	PRJNA591966	SRR10537899
18V3064F1	2018	Chicken	C. coli	PRJNA591966	SRR10537864
18V3081F1	2018	Chicken	C. coli	PRJNA591966	SRR10537841
19N3035F1	2019	Chicken	C. coli	PRJNA591966	SRR10537620
19N3126F1	2019	Chicken	C. coli	PRJNA591966	SRR10537599
17N3001F1	2017	Chicken	C. coli	PRJNA591966	SRR10537820
17N3046F1	2017	Chicken	C. coli	PRJNA591966	SRR10537828
17N3048F1	2017	Chicken	C. coli	PRJNA591966	SRR10537826
17Q3028F1	2017	Chicken	C. coli	PRJNA591966	SRR10537558
17Q3030F1	2017	Chicken	C. coli	PRJNA591966	SRR10537555
17Q3032F1	2017	Chicken	C. coli	PRJNA591966	SRR10537553
17Q3043F1	2017	Chicken	C. coli	PRJNA591966	SRR10537542
17Q3058F1	2017	Chicken	C. coli	PRJNA591966	SRR10537527
17Q3065F1	2017	Chicken	C. coli	PRJNA591966	SRR10537519
17Q3115F1	2017	Chicken	C. coli	PRJNA591966	SRR10537459
17V3006F1	2017	Chicken	C. coli	PRJNA591966	SRR10537991
17V3013F1	2017	Chicken	C. coli	PRJNA591966	SRR10537982
18A3010F1	2018	Chicken	C. coli	PRJNA591966	SRR10537734

18A3010F2	2018	Chicken	C. coli	PRJNA591966	SRR10537723
18A3013F1	2018	Chicken	C. coli	PRJNA591966	SRR10537690
18A3018F1	2018	Chicken	C. coli	PRJNA591966	SRR10537634
18A3019F1	2018	Chicken	C. coli	PRJNA591966	SRR10537623
18A3027F1	2018	Chicken	C. coli	PRJNA591966	SRR10537523
18N3012F1	2018	Chicken	C. coli	PRJNA591966	SRR10537709
18N3127F1	2018	Chicken	C. coli	PRJNA591966	SRR10537636
18N3135F1	2018	Chicken	C. coli	PRJNA591966	SRR10537631
18Q3040F1	2018	Chicken	C. coli	PRJNA591966	SRR10538005
18V3010F1	2018	Chicken	C. coli	PRJNA591966	SRR10537924
18V3013F1	2018	Chicken	C. coli	PRJNA591966	SRR10537922
18V3016F1	2018	Chicken	C. coli	PRJNA591966	SRR10537919
18V3016F2	2018	Chicken	C. coli	PRJNA591966	SRR10537917
18V3017F1	2018	Chicken	C. coli	PRJNA591966	SRR10537916
18V3018F1	2018	Chicken	C. coli	PRJNA591966	SRR10537915
18V3023F1	2018	Chicken	C. coli	PRJNA591966	SRR10537910
18V3026F1	2018	Chicken	C. coli	PRJNA591966	SRR10537906
18V3037F1	2018	Chicken	C. coli	PRJNA591966	SRR10537894
18V3038F2	2018	Chicken	C. coli	PRJNA591966	SRR10537892
18V3039F1	2018	Chicken	C. coli	PRJNA591966	SRR10537891
18V3041F1	2018	Chicken	C. coli	PRJNA591966	SRR10537889
18V3053F1	2018	Chicken	C. coli	PRJNA591966	SRR10537876
18V3056F1	2018	Chicken	C. coli	PRJNA591966	SRR10537872
18V3058F1	2018	Chicken	C. coli	PRJNA591966	SRR10537870
18V3060F1	2018	Chicken	C. coli	PRJNA591966	SRR10537868
18V3067F1	2018	Chicken	C. coli	PRJNA591966	SRR10537860
18V3071F2	2018	Chicken	C. coli	PRJNA591966	SRR10537854
18V3076F1	2018	Chicken	C. coli	PRJNA591966	SRR10537846
18V3077F1	2018	Chicken	C. coli	PRJNA591966	SRR10537845
18V3080F1	2018	Chicken	C. coli	PRJNA591966	SRR10537842
18V3082F1	2018	Chicken	C. coli	PRJNA591966	SRR10537839
18V3086F1	2018	Chicken	C. coli	PRJNA591966	SRR10537835
19N3119F1	2019	Chicken	C. coli	PRJNA591966	SRR10537602
17N3259F1	2017	Chicken	C. coli	PRJNA591966	SRR10537720
18A3004F1	2018	Chicken	C. coli	PRJNA591966	SRR10537457
18A3004F2	2018	Chicken	C. coli	PRJNA591966	SRR10537963
17V3002F1	2017	Chicken	C. coli	PRJNA591966	SRR10537994
17V3014F1	2017	Chicken	C. coli	PRJNA591966	SRR10537981
18A3005F1	2018	Chicken	C. coli	PRJNA591966	SRR10537852
18A3005F2	2018	Chicken	C. coli	PRJNA591966	SRR10537819
18A3059F1	2018	Chicken	C. coli	PRJNA591966	SRR10537918
18V3001F1	2018	Chicken	C. coli	PRJNA591966	SRR10537933
18V3068F1	2018	Chicken	C. coli	PRJNA591966	SRR10537859
17N3044F1	2017	Chicken	C. coli	PRJNA591966	SRR10537792
17N3227F1	2017	Chicken	C. coli	PRJNA591966	SRR10537735
17N3251F1	2017	Chicken	C. coli	PRJNA591966	SRR10537724
17Q3037F1	2017	Chicken	C. coli	PRJNA591966	SRR10537548
17Q3038F1	2017	Chicken	C. coli	PRJNA591966	SRR10537547
17Q3077F1	2017	Chicken	C. coli	PRJNA591966	SRR10537506
17Q3138F1	2017	Chicken	C. coli	PRJNA591966	SRR10538052
18A3060F1	2018	Chicken	C. coli	PRJNA591966	SRR10537907

18A3060F2	2018	Chicken	C. coli	PRJNA591966	SRR10537896
18N3001F1	2018	Chicken	C. coli	PRJNA591966	SRR10537718
18N3051F1	2018	Chicken	C. coli	PRJNA591966	SRR10537682
18Q3014F1	2018	Chicken	C. coli	PRJNA591966	SRR10538033
18Q3023F1	2018	Chicken	C. coli	PRJNA591966	SRR10538024
18V3003F1	2018	Chicken	C. coli	PRJNA591966	SRR10537931
18V3044F1	2018	Chicken	C. coli	PRJNA591966	SRR10537886
19N3195F1	2019	Chicken	C. coli	PRJNA591966	SRR10537589
17N3047F1	2017	Chicken	C. coli	PRJNA591966	SRR10537827
17N3067F1	2017	Chicken	C. coli	PRJNA591966	SRR10537780
17N3219F1	2017	Chicken	C. coli	PRJNA591966	SRR10537737
17Q3031F1	2017	Chicken	C. coli	PRJNA591966	SRR10537554
17Q3075F1	2017	Chicken	C. coli	PRJNA591966	SRR10537508
17Q3083F2	2017	Chicken	C. coli	PRJNA591966	SRR10537498
17Q3112F1	2017	Chicken	C. coli	PRJNA591966	SRR10537462
17Q3119F1	2017	Chicken	C. coli	PRJNA591966	SRR10538070
18A3002F1	2018	Chicken	C. coli	PRJNA591966	SRR10537790
18A3002F2	2018	Chicken	C. coli	PRJNA591966	SRR10537679
18A3003F1	2018	Chicken	C. coli	PRJNA591966	SRR10537568
18A3006F1	2018	Chicken	C. coli	PRJNA591966	SRR10537808
18A3009F1	2018	Chicken	C. coli	PRJNA591966	SRR10537756
18A3009F2	2018	Chicken	C. coli	PRJNA591966	SRR10537745
18A3022F1	2018	Chicken	C. coli	PRJNA591966	SRR10537590
18A3022F2	2018	Chicken	C. coli	PRJNA591966	SRR10537579
18N3030F1	2018	Chicken	C. coli	PRJNA591966	SRR10537694
18N3074F1	2018	Chicken	C. coli	PRJNA591966	SRR10537660
18N3084F1	2018	Chicken	C. coli	PRJNA591966	SRR10537649
18N3086F1	2018	Chicken	C. coli	PRJNA591966	SRR10537647
18N3123F1	2018	Chicken	C. coli	PRJNA591966	SRR10537637
18N3128F1	2018	Chicken	C. coli	PRJNA591966	SRR10537635
18N3129F1	2018	Chicken	C. coli	PRJNA591966	SRR10537633
18N3133F1	2018	Chicken	C. coli	PRJNA591966	SRR10537632
19N3022F1	2019	Chicken	C. coli	PRJNA591966	SRR10537622
19N3115F1	2019	Chicken	C. coli	PRJNA591966	SRR10537604
18V3027F1	2018	Chicken	C. coli	PRJNA591966	SRR10537905
18V3030F1	2018	Chicken	C. coli	PRJNA591966	SRR10537902
17N3093F1	2017	Chicken	C. coli	PRJNA591966	SRR10537763
17N3110F1	2017	Chicken	C. coli	PRJNA591966	SRR10537761
17Q3089F1	2017	Chicken	C. coli	PRJNA591966	SRR10537486
17Q3089F2	2017	Chicken	C. coli	PRJNA591966	SRR10537485
18V3019F1	2018	Chicken	C. coli	PRJNA591966	SRR10537914
17Q3081F1	2017	Chicken	C. coli	PRJNA591966	SRR10537502
18V3005F1	2018	Chicken	C. coli	PRJNA591966	SRR10537928
17N3015F1	2017	Chicken	C. coli	PRJNA591966	SRR10537814
17N3049F1	2017	Chicken	C. coli	PRJNA591966	SRR10537825
17N3079F1	2017	Chicken	C. coli	PRJNA591966	SRR10537771
17N3143F1	2017	Chicken	C. coli	PRJNA591966	SRR10537754
17N3146F1	2017	Chicken	C. coli	PRJNA591966	SRR10537753
17N3172F1	2017	Chicken	C. coli	PRJNA591966	SRR10537748
17N3180F1	2017	Chicken	C. coli	PRJNA591966	SRR10537744
17N3240F1	2017	Chicken	C. coli	PRJNA591966	SRR10537733
17N3243F1	2017	Chicken	C. coli	PRJNA591966	SRR10537730
17N3246F1	2017	Chicken	C. coli	PRJNA591966	SRR10537728
17N3248F1	2017	Chicken	C. coli	PRJNA591966	SRR10537726
17Q3025F1	2017	Chicken	C. coli	PRJNA591966	SRR10537561
17Q3026F1	2017	Chicken	C. coli	PRJNA591966	SRR10537560
17Q3036F1	2017	Chicken	C. coli	PRJNA591966	SRR10537549
17Q3042F1	2017	Chicken	C. coli	PRJNA591966	SRR10537543
17Q3105F1	2017	Chicken	C. coli	PRJNA591966	SRR10537469
17Q3106F1	2017	Chicken	C. coli	PRJNA591966	SRR10537467
17Q3137F1	2017	Chicken	C. coli	PRJNA591966	SRR10538053
17V3011F1	2017	Chicken	C. coli	PRJNA591966	SRR10537986
17V3012F1	2017	Chicken	C. coli	PRJNA591966	SRR10537984
17V3019F1	2017	Chicken	C. coli	PRJNA591966	SRR10537976
17V3039F1	2017	Chicken	C. coli	PRJNA591966	SRR10537955
17V3048F1	2017	Chicken	C. coli	PRJNA591966	SRR10537947
17V3055F2	2017	Chicken	C. coli	PRJNA591966	SRR10537942
17V3058F1	2017	Chicken	C. coli	PRJNA591966	SRR10537938
17V3059F1	2017	Chicken	C. coli	PRJNA591966	SRR10537936
18A3065F1	2018	Chicken	C. coli	PRJNA591966	SRR10537832
18A3065F2	2018	Chicken	C. coli	PRJNA591966	SRR10537831
18N3055F1	2018	Chicken	C. coli	PRJNA591966	SRR10537677
18N3067F1	2018	Chicken	C. coli	PRJNA591966	SRR10537668
18N3083F1	2018	Chicken	C. coli	PRJNA591966	SRR10537650
18N3087F1	2018	Chicken	C. coli	PRJNA591966	SRR10537646
18N3088F1	2018	Chicken	C. coli	PRJNA591966	SRR10537644
18N3093F1	2018	Chicken	C. coli	PRJNA591966	SRR10537639
18V3006F1	2018	Chicken	C. coli	PRJNA591966	SRR10537927
18V3022F1	2018	Chicken	C. coli	PRJNA591966	SRR10537911
18V3035F1	2018	Chicken	C. coli	PRJNA591966	SRR10537897
18V3045F1	2018	Chicken	C. coli	PRJNA591966	SRR10537884
18V3049F1	2018	Chicken	C. coli	PRJNA591966	SRR10537880
18V3057F1	2018	Chicken	C. coli	PRJNA591966	SRR10537871
18V3066F1	2018	Chicken	C. coli	PRJNA591966	SRR10537862
18V3072F1	2018	Chicken	C. coli	PRJNA591966	SRR10537853
18V3073F2	2018	Chicken	C. coli	PRJNA591966	SRR10537849
18V3075F1	2018	Chicken	C. coli	PRJNA591966	SRR10537847
19N3082F1	2019	Chicken	C. coli	PRJNA591966	SRR10537617
19N3099F1	2019	Chicken	C. coli	PRJNA591966	SRR10537615
19N3106F1	2019	Chicken	C. coli	PRJNA591966	SRR10537611
18A3063F2	2018	Chicken	C. coli	PRJNA591966	SRR10537851
17Q3050F1	2017	Chicken	C. coli	PRJNA591966	SRR10537535
17Q3085F1	2017	Chicken	C. coli	PRJNA591966	SRR10537495
18V3031F1	2018	Chicken	C. coli	PRJNA591966	SRR10537901
17N3022F1	2017	Chicken	C. coli	PRJNA591966	SRR10537809
18V3042F1	2018	Chicken	C. coli	PRJNA591966	SRR10537888
17Q3022F1	2017	Chicken	C. coli	PRJNA591966	SRR10537564
17Q3033F1	2017	Chicken	C. coli	PRJNA591966	SRR10537552
17Q3068F1	2017	Chicken	C. coli	PRJNA591966	SRR10537516
17Q3078F1	2017	Chicken	C. coli	PRJNA591966	SRR10537505
17Q3079F1	2017	Chicken	C. coli	PRJNA591966	SRR10537504
17Q3086F2	2017	Chicken	C. coli	PRJNA591966	SRR10537492

17Q3116F1	2017	Chicken	C. coli	PRJNA591966	SRR10537458
17Q3126F1	2017	Chicken	C. coli	PRJNA591966	SRR10538063
18A3046F1	2018	Chicken	C. coli	PRJNA591966	SRR10538051
18Q3026F1	2018	Chicken	C. coli	PRJNA591966	SRR10538021
18Q3028F1	2018	Chicken	C. coli	PRJNA591966	SRR10538019
18Q3033F1	2018	Chicken	C. coli	PRJNA591966	SRR10538013
18Q3036F1	2018	Chicken	C. coli	PRJNA591966	SRR10538010
18N3052F1	2018	Chicken	C. coli	PRJNA591966	SRR10537681
17N3021F1	2017	Chicken	C. coli	PRJNA591966	SRR10537810
17N3068F1	2017	Chicken	C. coli	PRJNA591966	SRR10537779
17N3084F1	2017	Chicken	C. coli	PRJNA591966	SRR10537769
17N3092F1	2017	Chicken	C. coli	PRJNA591966	SRR10537764
17Q3024F1	2017	Chicken	C. coli	PRJNA591966	SRR10537562
17Q3041F1	2017	Chicken	C. coli	PRJNA591966	SRR10537544
17V3020F1	2017	Chicken	C. coli	PRJNA591966	SRR10537975
18N3017F1	2018	Chicken	C. coli	PRJNA591966	SRR10537705
18N3045F1	2018	Chicken	C. coli	PRJNA591966	SRR10537686
18Q3024F1	2018	Chicken	C. coli	PRJNA591966	SRR10538023
18V3085F1	2018	Chicken	C. coli	PRJNA591966	SRR10537837
19N3149F1	2019	Chicken	C. coli	PRJNA591966	SRR10537597
17N3151F1	2017	Chicken	C. coli	PRJNA591966	SRR10537752
17N3241F1	2017	Chicken	C. coli	PRJNA591966	SRR10537732
17N3245F1	2017	Chicken	C. coli	PRJNA591966	SRR10537729
17N3247F1	2017	Chicken	C. coli	PRJNA591966	SRR10537727
17Q3059F1	2017	Chicken	C. coli	PRJNA591966	SRR10537526
17Q3103F1	2017	Chicken	C. coli	PRJNA591966	SRR10537471
17Q3104F1	2017	Chicken	C. coli	PRJNA591966	SRR10537470
17Q3110F2	2017	Chicken	C. coli	PRJNA591966	SRR10537464
17V3021F1	2017	Chicken	C. coli	PRJNA591966	SRR10537973
18N3028F1	2018	Chicken	C. coli	PRJNA591966	SRR10537696
18N3032F1	2018	Chicken	C. coli	PRJNA591966	SRR10537692
18N3068F1	2018	Chicken	C. coli	PRJNA591966	SRR10537666
18Q3029F1	2018	Chicken	C. coli	PRJNA591966	SRR10538017
19N3014F1	2019	Chicken	C. coli	PRJNA591966	SRR10537626
19N3188F1	2019	Chicken	C. coli	PRJNA591966	SRR10537594
18A3001F1	2018	Chicken	C. coli	PRJNA591966	SRR10537791
18N3072F1	2018	Chicken	C. coli	PRJNA591966	SRR10537662
19N3114F1	2019	Chicken	C. coli	PRJNA591966	SRR10537605
19N3152F1	2019	Chicken	C. coli	PRJNA591966	SRR10537596
17N3206F1	2017	Chicken	C. coli	PRJNA591966	SRR10537740
17N3066F1	2017	Chicken	C. coli	PRJNA591966	SRR10537781
17N3119F1	2017	Chicken	C. coli	PRJNA591966	SRR10537760
17Q3027F1	2017	Chicken	C. coli	PRJNA591966	SRR10537559
17Q3034F1	2017	Chicken	C. coli	PRJNA591966	SRR10537551
17Q3096F1	2017	Chicken	C. coli	PRJNA591966	SRR10537478
18A3014F1	2018	Chicken	C. coli	PRJNA591966	SRR10537678
18N3024F1	2018	Chicken	C. coli	PRJNA591966	SRR10537700
18N3147F1	2018	Chicken	C. coli	PRJNA591966	SRR10537629
19N3017F1	2019	Chicken	C. coli	PRJNA591966	SRR10537625
19N3021F1	2019	Chicken	C. coli	PRJNA591966	SRR10537624
19N3057F1	2019	Chicken	C. coli	PRJNA591966	SRR10537618

19N3121F1	2019	Chicken	C. coli	PRJNA591966	SRR10537600
17N3024F1	2017	Chicken	C. coli	PRJNA591966	SRR10537806
17N3071F1	2017	Chicken	C. coli	PRJNA591966	SRR10537776
17N3181F1	2017	Chicken	C. coli	PRJNA591966	SRR10537743
17Q3035F1	2017	Chicken	C. coli	PRJNA591966	SRR10537550
17Q3044F1	2017	Chicken	C. coli	PRJNA591966	SRR10537541
17Q3045F1	2017	Chicken	C. coli	PRJNA591966	SRR10537540
17Q3046F1	2017	Chicken	C. coli	PRJNA591966	SRR10537539
17Q3047F1	2017	Chicken	C. coli	PRJNA591966	SRR10537538
17Q3052F1	2017	Chicken	C. coli	PRJNA591966	SRR10537533
17Q3070F1	2017	Chicken	C. coli	PRJNA591966	SRR10537514
17Q3087F2	2017	Chicken	C. coli	PRJNA591966	SRR10537489
17V3007F1	2017	Chicken	C. coli	PRJNA591966	SRR10537990
17V3031F2	2017	Chicken	C. coli	PRJNA591966	SRR10537965
18N3080F1	2018	Chicken	C. coli	PRJNA591966	SRR10537653
17N3029F1	2017	Lamb	C. coli	PRJNA591966	SRR10537802
17Q3009F1	2017	Lamb	C. coli	PRJNA591966	SRR10537580
17Q3021F1	2017	Lamb	C. coli	PRJNA591966	SRR10537565
17Q3144F1	2017	Lamb	C. coli	PRJNA591966	SRR10538046
18N3007F1	2018	Lamb	C. coli	PRJNA591966	SRR10537713
18N3059F1	2018	Lamb	C. coli	PRJNA591966	SRR10537674
18Q3004F1	2018	Lamb	C. coli	PRJNA591966	SRR10538043
18N3085F1	2018	Lamb	C. coli	PRJNA591966	SRR10537648
18N3138F1	2018	Lamb	C. coli	PRJNA591966	SRR10537630
17Q3015F1	2017	Lamb	C. coli	PRJNA591966	SRR10537573
18N3089F1	2018	Lamb	C. coli	PRJNA591966	SRR10537643
19N3109F1	2019	Lamb	C. coli	PRJNA591966	SRR10537609
18N3015F1	2018	Pork	C. coli	PRJNA591966	SRR10537706
18N3019F1	2018	Pork	C. coli	PRJNA591966	SRR10537704
17N3050F1	2017	Pork	C. coli	PRJNA591966	SRR10537824
18N3079F1	2018	Pork	C. coli	PRJNA591966	SRR10537654
18Q3052F1	2018	Pork	C. coli	PRJNA591966	SRR10537997
17N3207F1	2017	Pork	C. coli	PRJNA591966	SRR10537739
17N3153F1	2017	Pork	C. coli	PRJNA591966	SRR10537751
18N3048F1	2018	Pork	C. coli	PRJNA591966	SRR10537684
18Q3008F1	2018	Pork	C. coli	PRJNA591966	SRR10538039
18Q3009F1	2018	Pork	C. coli	PRJNA591966	SRR10538038
18N3075F1	2018	Pork	C. coli	PRJNA591966	SRR10537659
18N3029F1	2018	Pork	C. coli	PRJNA591966	SRR10537695
18Q3043F2	2018	Pork	C. coli	PRJNA591966	SRR10538001
17N3138F1	2017	Pork	C. coli	PRJNA591966	SRR10537757
17N3257F1	2017	Pork	C. coli	PRJNA591966	SRR10537722
17N3184F1	2017	Pork	C. coli	PRJNA591966	SRR10537742
17N3154F1	2017	Pork	C. coli	PRJNA591966	SRR10537750
17N3215F1	2017	Pork	C. coli	PRJNA591966	SRR10537738
18A3052F1	2018	Pork	C. coli	PRJNA591966	SRR10537996
18A3052F2	2018	Pork	C. coli	PRJNA591966	SRR10537985
18N3034F1	2018	Pork	C. coli	PRJNA591966	SRR10537689
18N3061F1	2018	Pork	C. coli	PRJNA591966	SRR10537673
18Q3042F1	2018	Pork	C. coli	PRJNA591966	SRR10538003
18Q3044F1	2018	Pork	C. coli	PRJNA591966	SRR10538000

19N3190F1	2019	Pork	C. coli	PRJNA591966	SRR10537593
18A3057F1	2018	Chicken	C. coli	PRJNA591966	SRR10537929
18A3062F1	2018	Chicken	C. coli	PRJNA591966	SRR10537874
18A3063F1	2018	Chicken	C. coli	PRJNA591966	SRR10537863
18N3082F1	2018	Chicken	C. coli	PRJNA591966	SRR10537651
17Q3023F1	2017	Chicken	C. coli	PRJNA591966	SRR10537563
17Q3073F1	2017	Chicken	C. coli	PRJNA591966	SRR10537510
17Q3053F1	2017	Chicken	C. coli	PRJNA591966	SRR10537532
17Q3099F1	2017	Chicken	C. coli	PRJNA591966	SRR10537475
18Q3018F1	2018	Chicken	C. coli	PRJNA591966	SRR10538028
18Q3019F1	2018	Chicken	C. coli	PRJNA591966	SRR10538027
17V3023F1	2017	Chicken	C. coli	PRJNA591966	SRR10537971
17N3018F1	2017	Chicken	C. coli	PRJNA591966	SRR10537812
17Q3076F1	2017	Chicken	C. coli	PRJNA591966	SRR10537507
17Q3084F2	2017	Chicken	C. coli	PRJNA591966	SRR10537496
17Q3129F1	2017	Chicken	C. coli	PRJNA591966	SRR10538060
18A3055F1	2018	Chicken	C. coli	PRJNA591966	SRR10537951
18N3006F1	2018	Chicken	C. coli	PRJNA591966	SRR10537714
18N3027F1	2018	Chicken	C. coli	PRJNA591966	SRR10537697
18V3029F1	2018	Chicken	C. coli	PRJNA591966	SRR10537903
18V3034F1	2018	Chicken	C. coli	PRJNA591966	SRR10537898
17N3051F1	2017	Pork	C. coli	PRJNA591966	SRR10537788
17N3260F1	2017	Pork	C. coli	PRJNA591966	SRR10537719
18Q3007F1	2018	Pork	C. coli	PRJNA591966	SRR10538041
17N3033F1	2017	Beef	C. jejuni	PRJNA591966	SRR10537798
17Q3133F1	2017	Lamb	C. jejuni	PRJNA591966	SRR10538057
18A3017F1	2018	Chicken	C. jejuni	PRJNA591966	SRR10537645
18A3023F1	2018	Chicken	C. jejuni	PRJNA591966	SRR10537567
18A3024F1	2018	Chicken	C. jejuni	PRJNA591966	SRR10537556
18A3035F1	2018	Chicken	C. jejuni	PRJNA591966	SRR10537468
18A3068F1	2018	Chicken	C. jejuni	PRJNA591966	SRR10537823
18N3065F1	2018	Lamb	C. jejuni	PRJNA591966	SRR10537670
18Q3043F1	2018	Pork	C. jejuni	PRJNA591966	SRR10538002
17Q3007F1	2017	Beef	C. jejuni	PRJNA591966	SRR10537582
17Q3008F1	2017	Beef	C. jejuni	PRJNA591966	SRR10537581
18N3026F1	2018	Lamb	C. jejuni	PRJNA591966	SRR10537698
18N3033F1	2018	Lamb	C. jejuni	PRJNA591966	SRR10537691
18N3056F1	2018	Lamb	C. jejuni	PRJNA591966	SRR10537676
18N3069F1	2018	Chicken	C. jejuni	PRJNA591966	SRR10537665
18N3081F1	2018	Pork	C. jejuni	PRJNA591966	SRR10537652
18V3071F1	2018	Chicken	C. jejuni	PRJNA591966	SRR10537855
17Q3049F1	2017	Chicken	C. jejuni	PRJNA591966	SRR10537536
17Q3072F1	2017	Chicken	C. jejuni	PRJNA591966	SRR10537511
17Q3122F1	2017	Chicken	C. jejuni	PRJNA591966	SRR10538067
17V3030F1	2017	Chicken	C. jejuni	PRJNA591966	SRR10537967
17V3055F1	2017	Chicken	C. jejuni	PRJNA591966	SRR10537943
17V3060F1	2017	Chicken	C. jejuni	PRJNA591966	SRR10537935
18N3054F1	2018	Chicken	C. jejuni	PRJNA591966	SRR10537680
18V3008F1	2018	Chicken	C. jejuni	PRJNA591966	SRR10537926
18V3070F1	2018	Chicken	C. jejuni	PRJNA591966	SRR10537856
17N3065F1	2017	Chicken	C. jejuni	PRJNA591966	SRR10537782
17Q3060F1	2017	Chicken	C. jejuni	PRJNA591966	SRR10537525
18N3153F1	2018	Chicken	C. jejuni	PRJNA591966	SRR10537627
18Q3021F1	2018	Chicken	C. jejuni	PRJNA591966	SRR10538025
18V3085F2	2018	Chicken	C. jejuni	PRJNA591966	SRR10537836
19N3083F1	2019	Chicken	C. jejuni	PRJNA591966	SRR10537616
19N3111F1	2019	Pork	C. jejuni	PRJNA591966	SRR10537607
17N3105F1	2017	Chicken	C. jejuni	PRJNA591966	SRR10537762
17V3029F1	2017	Chicken	C. jejuni	PRJNA591966	SRR10537968
18A3011F1	2018	Chicken	C. jejuni	PRJNA591966	SRR10537712
18A3012F1	2018	Lamb	C. jejuni	PRJNA591966	SRR10537701
18V3078F1	2018	Chicken	C. jejuni	PRJNA591966	SRR10537844
17N3027F1	2017	Chicken	C. jejuni	PRJNA591966	SRR10537804
17Q3111F1	2017	Chicken	C. jejuni	PRJNA591966	SRR10537463
17Q3127F1	2017	Chicken	C. jejuni	PRJNA591966	SRR10538061
17V3012F2	2017	Chicken	C. jejuni	PRJNA591966	SRR10537983
17V3033F1	2017	Chicken	C. jejuni	PRJNA591966	SRR10537961
17N3042F1	2017	Lamb	C. jejuni	PRJNA591966	SRR10537794
17N3053F1	2017	Lamb	C. jejuni	PRJNA591966	SRR10537787
17N3062F1	2017	Beef	C. jejuni	PRJNA591966	SRR10537783
17N3070F1	2017	Lamb	C. jejuni	PRJNA591966	SRR10537777
17N3080F1	2017	Beef	C. jejuni	PRJNA591966	SRR10537770
17N3175F1	2017	Chicken	C. jejuni	PRJNA591966	SRR10537747
17N3205F1	2017	Chicken	C. jejuni	PRJNA591966	SRR10537741
17Q3001F4	2017	Beef	C. jejuni	PRJNA591966	SRR10537588
17Q3012F1	2017	Lamb	C. jejuni	PRJNA591966	SRR10537576
17Q3014F1	2017	Lamb	C. jejuni	PRJNA591966	SRR10537574
17Q3018F1	2017	Lamb	C. jejuni	PRJNA591966	SRR10537570
17Q3056F1	2017	Chicken	C. jejuni	PRJNA591966	SRR10537529
17Q3062F1	2017	Chicken	C. jejuni	PRJNA591966	SRR10537522
17Q3066F1	2017	Chicken	C. jejuni	PRJNA591966	SRR10537518
17Q3071F1	2017	Chicken	C. jejuni	PRJNA591966	SRR10537513
17Q3074F1	2017	Chicken	C. jejuni	PRJNA591966	SRR10537509
17Q3082F1	2017	Chicken	C. jejuni	PRJNA591966	SRR10537500
17Q3092F1	2017	Lamb	C. jejuni	PRJNA591966	SRR10537483
17Q3093F1	2017	Lamb	C. jejuni	PRJNA591966	SRR10537482
17Q3097F1	2017	Chicken	C. jejuni	PRJNA591966	SRR10537477
17Q3102F1	2017	Chicken	C. jejuni	PRJNA591966	SRR10537472
17Q3108F1	2017	Chicken	C. jejuni	PRJNA591966	SRR10537466
17Q3113F1	2017	Chicken	C. jejuni	PRJNA591966	SRR10537461
17Q3114F2	2017	Chicken	C. jejuni	PRJNA591966	SRR10537460
17Q3136F1	2017	Chicken	C. jejuni	PRJNA591966	SRR10538054
18A3015F1	2018	Lamb	C. jejuni	PRJNA591966	SRR10537667
18A3021F1	2018	Chicken	C. jejuni	PRJNA591966	SRR10537601
18A3025F1	2018	Chicken	C. jejuni	PRJNA591966	SRR10537545
18A3047F1	2018	Lamb	C. jejuni	PRJNA591966	SRR10538040
18A3048F1	2018	Lamb	C. jejuni	PRJNA591966	SRR10538029
18A3050F1	2018	Lamb	C. jejuni	PRJNA591966	SRR10538007
18A3064F1	2018	Lamb	C. jejuni	PRJNA591966	SRR10537840
18N3002F1	2018	Lamb	C. jejuni	PRJNA591966	SRR10537717
18N3013F1	2018	Pork	C. jejuni	PRJNA591966	SRR10537708
18N3022F1	2018	Lamb	C. jejuni	PRJNA591966	SRR10537703

18N3023F1	2018	Lamb	C. jejuni	PRJNA591966	SRR10537702
18N3050F1	2018	Chicken	C. jejuni	PRJNA591966	SRR10537683
18N3090F1	2018	Pork	C. jejuni	PRJNA591966	SRR10537642
18N3148F1	2018	Lamb	C. jejuni	PRJNA591966	SRR10537628
18Q3010F1	2018	Pork	C. jejuni	PRJNA591966	SRR10538037
18Q3011F1	2018	Pork	C. jejuni	PRJNA591966	SRR10538036
18Q3012F1	2018	Chicken	C. jejuni	PRJNA591966	SRR10538035
18Q3020F1	2018	Chicken	C. jejuni	PRJNA591966	SRR10538026
18Q3030F1	2018	Chicken	C. jejuni	PRJNA591966	SRR10538016
18Q3034F1	2018	Chicken	C. jejuni	PRJNA591966	SRR10538012
18V3025F1	2018	Chicken	C. jejuni	PRJNA591966	SRR10537908
18V3038F1	2018	Chicken	C. jejuni	PRJNA591966	SRR10537893
18V3046F1	2018	Chicken	C. jejuni	PRJNA591966	SRR10537883
19N3103F1	2019	Chicken	C. jejuni	PRJNA591966	SRR10537614
19N3105F1	2019	Beef	C. jejuni	PRJNA591966	SRR10537613
19N3116F1	2019	Pork	C. jejuni	PRJNA591966	SRR10537603
17N3055F1	2017	Chicken	C. jejuni	PRJNA591966	SRR10537786
17N3072F1	2017	Chicken	C. jejuni	PRJNA591966	SRR10537775
17V3028F1	2017	Chicken	C. jejuni	PRJNA591966	SRR10537969
18A3007F1	2018	Chicken	C. jejuni	PRJNA591966	SRR10537797
18A3007F2	2018	Chicken	C. jejuni	PRJNA591966	SRR10537789
18A3054F1	2018	Chicken	C. jejuni	PRJNA591966	SRR10537962
18N3094F1	2018	Chicken	C. jejuni	PRJNA591966	SRR10537638
19N3191F1	2019	Chicken	C. jejuni	PRJNA591966	SRR10537592
17N3031F1	2017	Beef	C. jejuni	PRJNA591966	SRR10537800
17Q3004F1	2017	Beef	C. jejuni	PRJNA591966	SRR10537585
17Q3016F1	2017	Lamb	C. jejuni	PRJNA591966	SRR10537572
17Q3057F1	2017	Chicken	C. jejuni	PRJNA591966	SRR10537528
18A3029F1	2018	Chicken	C. jejuni	PRJNA591966	SRR10537501
18A3030F1	2018	Chicken	C. jejuni	PRJNA591966	SRR10537490
18V3024F1	2018	Chicken	C. jejuni	PRJNA591966	SRR10537909
19N3185F1	2019	Chicken	C. jejuni	PRJNA591966	SRR10537595
17N3016F1	2017	Lamb	C. jejuni	PRJNA591966	SRR10537813
17N3090F1	2017	Chicken	C. jejuni	PRJNA591966	SRR10537766
17N3155F1	2017	Beef	C. jejuni	PRJNA591966	SRR10537749
17Q3002F1	2017	Beef	C. jejuni	PRJNA591966	SRR10537587
17Q3005F1	2017	Beef	C. jejuni	PRJNA591966	SRR10537584
17Q3010F1	2017	Lamb	C. jejuni	PRJNA591966	SRR10537578
17Q3011F1	2017	Lamb	C. jejuni	PRJNA591966	SRR10537577
17Q3013F1	2017	Lamb	C. jejuni	PRJNA591966	SRR10537575
17Q3017F1	2017	Lamb	C. jejuni	PRJNA591966	SRR10537571
17Q3039F1	2017	Chicken	C. jejuni	PRJNA591966	SRR10537546
17Q3090F1	2017	Beef	C. jejuni	PRJNA591966	SRR10537484
17Q3132F1	2017	Beef	C. jejuni	PRJNA591966	SRR10538058
17Q3135F1	2017	Lamb	C. jejuni	PRJNA591966	SRR10538055
18A3016F1	2018	Beef	C. jejuni	PRJNA591966	SRR10537656
18A3032F1	2018	Lamb	C. jejuni	PRJNA591966	SRR10537479
18A3049F1	2018	Beef	C. jejuni	PRJNA591966	SRR10538018
18A3067F1	2018	Lamb	C. jejuni	PRJNA591966	SRR10537829
18N3014F1	2018	Lamb	C. jejuni	PRJNA591966	SRR10537707
18N3057F1	2018	Beef	C. jejuni	PRJNA591966	SRR10537675

18N3064F1	2018	Lamb	C. jejuni	PRJNA591966	SRR10537671
18Q3002F1	2018	Beef	C. jejuni	PRJNA591966	SRR10538045
18Q3003F1	2018	Lamb	C. jejuni	PRJNA591966	SRR10538044
18V3066F2	2018	Chicken	C. jejuni	PRJNA591966	SRR10537861
18V3004F1	2018	Chicken	C. jejuni	PRJNA591966	SRR10537930
18V3061F1	2018	Chicken	C. jejuni	PRJNA591966	SRR10537867
18A3044F1	2018	Chicken	C. jejuni	PRJNA591966	SRR10537456
18V3036F2	2018	Chicken	C. jejuni	PRJNA591966	SRR10537895
18V3084F1	2018	Chicken	C. jejuni	PRJNA591966	SRR10537838
17Q3134F1	2017	Lamb	C. jejuni	PRJNA591966	SRR10538056
18N3036F1	2018	Lamb	C. jejuni	PRJNA591966	SRR10537688
18Q3005F1	2018	Lamb	C. jejuni	PRJNA591966	SRR10538042
18V3050F1	2018	Chicken	C. jejuni	PRJNA591966	SRR10537879
19N3110F1	2019	Lamb	C. jejuni	PRJNA591966	SRR10537608
17V3035F1	2017	Chicken	C. jejuni	PRJNA591966	SRR10537959
18V3043F1	2018	Chicken	C. jejuni	PRJNA591966	SRR10537887
17Q3087F1	2017	Chicken	C. jejuni	PRJNA591966	SRR10537491
17Q3101F1	2017	Chicken	C. jejuni	PRJNA591966	SRR10537473
17Q3121F1	2017	Chicken	C. jejuni	PRJNA591966	SRR10538068
18N3031F1	2018	Chicken	C. jejuni	PRJNA591966	SRR10537693
18N3037F1	2018	Beef	C. jejuni	PRJNA591966	SRR10537687
17N3014F1	2017	Chicken	C. jejuni	PRJNA591966	SRR10537815
17N3087F1	2017	Chicken	C. jejuni	PRJNA591966	SRR10537768
17N3131F1	2017	Chicken	C. jejuni	PRJNA591966	SRR10537758
17Q3048F1	2017	Chicken	C. jejuni	PRJNA591966	SRR10537537
17Q3055F1	2017	Chicken	C. jejuni	PRJNA591966	SRR10537530
17Q3061F1	2017	Chicken	C. jejuni	PRJNA591966	SRR10537524
17Q3080F1	2017	Chicken	C. jejuni	PRJNA591966	SRR10537503
17Q3120F1	2017	Chicken	C. jejuni	PRJNA591966	SRR10538069
17V3022F1	2017	Chicken	C. jejuni	PRJNA591966	SRR10537972
18Q3031F1	2018	Chicken	C. jejuni	PRJNA591966	SRR10538015
18Q3037F1	2018	Chicken	C. jejuni	PRJNA591966	SRR10538009
18Q3038F1	2018	Chicken	C. jejuni	PRJNA591966	SRR10538008
17N3013F1	2017	Chicken	C. jejuni	PRJNA591966	SRR10537816
17N3076F1	2017	Chicken	C. jejuni	PRJNA591966	SRR10537773
17N3177F1	2017	Chicken	C. jejuni	PRJNA591966	SRR10537746
17N3242F1	2017	Chicken	C. jejuni	PRJNA591966	SRR10537731
17Q3109F1	2017	Chicken	C. jejuni	PRJNA591966	SRR10537465
18N3066F1	2018	Chicken	C. jejuni	PRJNA591966	SRR10537669
18V3014F1	2018	Chicken	C. jejuni	PRJNA591966	SRR10537921
18V3068F2	2018	Chicken	C. jejuni	PRJNA591966	SRR10537858
18V3079F1	2018	Chicken	C. jejuni	PRJNA591966	SRR10537843
19N3023F1	2019	Chicken	C. jejuni	PRJNA591966	SRR10537621
19N3055F1	2019	Beef	C. jejuni	PRJNA591966	SRR10537619
17N3009F1	2017	Pork	C. jejuni	PRJNA591966	SRR10537818
17V3052F1	2017	Chicken	C. jejuni	PRJNA591966	SRR10537944
18V3054F1	2018	Chicken	C. jejuni	PRJNA591966	SRR10537875
18V3062F1	2018	Chicken	C. jejuni	PRJNA591966	SRR10537866
18V3063F1	2018	Chicken	C. jejuni	PRJNA591966	SRR10537865
18V4087F2	2018	Chicken	C. jejuni	PRJNA591966	SRR10537834
17Q3100F1	2017	Chicken	C. jejuni	PRJNA591966	SRR10537474

18N3008F1	2018	Pork	C. jejuni	PRJNA591966	SRR10537711
18N3009F1	2018	Lamb	C. jejuni	PRJNA591966	SRR10537710
18V3028F1	2018	Chicken	C. jejuni	PRJNA591966	SRR10537904
18V3048F1	2018	Chicken	C. jejuni	PRJNA591966	SRR10537881
17V3042F1	2017	Chicken	C. jejuni	PRJNA591966	SRR10537952
18A3028F1	2018	Chicken	C. jejuni	PRJNA591966	SRR10537512
18Q3027F1	2018	Chicken	C. jejuni	PRJNA591966	SRR10538020
17N3030F1	2017	Lamb	C. jejuni	PRJNA591966	SRR10537801
17Q3098F1	2017	Chicken	C. jejuni	PRJNA591966	SRR10537476
17V3015F1	2017	Chicken	C. jejuni	PRJNA591966	SRR10537980
17V3031F1	2017	Chicken	C. jejuni	PRJNA591966	SRR10537966
18Q3015F1	2018	Chicken	C. jejuni	PRJNA591966	SRR10538032
18V3047F1	2018	Chicken	C. jejuni	PRJNA591966	SRR10537882
17N3061F1	2017	Chicken	C. jejuni	PRJNA591966	SRR10537784
17N3073F1	2017	Chicken	C. jejuni	PRJNA591966	SRR10537774
18N3076F1	2018	Chicken	C. jejuni	PRJNA591966	SRR10537658
18N3073F1	2018	Chicken	C. jejuni	PRJNA591966	SRR10537661
17Q3067F1	2017	Chicken	C. jejuni	PRJNA591966	SRR10537517
17Q3084F1	2017	Chicken	C. jejuni	PRJNA591966	SRR10537497
18V3074F1	2018	Chicken	C. jejuni	PRJNA591966	SRR10537848
18Q3032F1	2018	Chicken	C. jejuni	PRJNA591966	SRR10538014
18A3056F1	2018	Chicken	C. jejuni	PRJNA591966	SRR10537940
18V3032F1	2018	Chicken	C. jejuni	PRJNA591966	SRR10537900
17N3258F1	2017	Chicken	C. jejuni	PRJNA591966	SRR10537721
17V3009F1	2017	Chicken	C. jejuni	PRJNA591966	SRR10537988
17V3032F1	2017	Chicken	C. jejuni	PRJNA591966	SRR10537964
18N3005F1	2018	Beef	C. jejuni	PRJNA591966	SRR10537715
18N3070F1	2018	Chicken	C. jejuni	PRJNA591966	SRR10537664
18N3077F1	2018	Chicken	C. jejuni	PRJNA591966	SRR10537657
18N3078F1	2018	Chicken	C. jejuni	PRJNA591966	SRR10537655
18Q3046F1	2018	Pork	C. jejuni	PRJNA591966	SRR10537999
18Q3047F1	2018	Pork	C. jejuni	PRJNA591966	SRR10537998
18V5088F3	2018	Chicken	C. jejuni	PRJNA591966	SRR10537833
17Q3083F1	2017	Chicken	C. jejuni	PRJNA591966	SRR10537499
17Q3088F1	2017	Chicken	C. jejuni	PRJNA591966	SRR10537488
17Q3088F2	2017	Chicken	C. jejuni	PRJNA591966	SRR10537487
17Q3095F1	2017	Chicken	C. jejuni	PRJNA591966	SRR10537480
17Q3139F1	2017	Chicken	C. jejuni	PRJNA591966	SRR10538050
17V3008F1	2017	Chicken	C. jejuni	PRJNA591966	SRR10537989
17V3018F1	2017	Chicken	C. jejuni	PRJNA591966	SRR10537977
17V3057F1	2017	Chicken	C. jejuni	PRJNA591966	SRR10537939
18A3069F1	2018	Chicken	C. jejuni	PRJNA591966	SRR10537822
18Q3035F1	2018	Chicken	C. jejuni	PRJNA591966	SRR10538011
18Q3039F1	2018	Chicken	C. jejuni	PRJNA591966	SRR10538006
18V3011F1	2018	Chicken	C. jejuni	PRJNA591966	SRR10537923
18V3021F1	2018	Chicken	C. jejuni	PRJNA591966	SRR10537912
17N3032F1	2017	Lamb	C. jejuni	PRJNA591966	SRR10537799
17N3034F1	2017	Beef	C. jejuni	PRJNA591966	SRR10537796
17N3035F1	2017	Lamb	C. jejuni	PRJNA591966	SRR10537795
17N3125F1	2017	Chicken	C. jejuni	PRJNA591966	SRR10537759
17Q3006F1	2017	Beef	C. jejuni	PRJNA591966	SRR10537583
17Q3019F1	2017	Lamb	C. jejuni	PRJNA591966	SRR10537569
17Q3020F1	2017	Lamb	C. jejuni	PRJNA591966	SRR10537566
17Q3142F1	2017	Chicken	C. jejuni	PRJNA591966	SRR10538047
17N3010F1	2017	Pork	C. jejuni	PRJNA591966	SRR10537817
17N3057F1	2017	Chicken	C. jejuni	PRJNA591966	SRR10537785
17Q3029F1	2017	Chicken	C. jejuni	PRJNA591966	SRR10537557
17Q3069F1	2017	Chicken	C. jejuni	PRJNA591966	SRR10537515
17Q3086F1	2017	Chicken	C. jejuni	PRJNA591966	SRR10537493
17Q3125F1	2017	Chicken	C. jejuni	PRJNA591966	SRR10538064
17Q3130F1	2017	Chicken	C. jejuni	PRJNA591966	SRR10538059
17Q3140F1	2017	Chicken	C. jejuni	PRJNA591966	SRR10538049
18N3004F1	2018	Chicken	C. jejuni	PRJNA591966	SRR10537716
18N3025F1	2018	Beef	C. jejuni	PRJNA591966	SRR10537699
18Q3041F1	2018	Pork	C. jejuni	PRJNA591966	SRR10538004
18V3069F1	2018	Chicken	C. jejuni	PRJNA591966	SRR10537857
18V3073F1	2018	Chicken	C. jejuni	PRJNA591966	SRR10537850
19N3192F1	2019	Chicken	C. jejuni	PRJNA591966	SRR10537591
17N3221F1	2017	Chicken	C. jejuni	PRJNA591966	SRR10537736
17Q3124F1	2017	Chicken	C. jejuni	PRJNA591966	SRR10538065
17V3058F2	2017	Chicken	C. jejuni	PRJNA591966	SRR10537937
18V3020F1	2018	Chicken	C. jejuni	PRJNA591966	SRR10537913
18V3040F1	2018	Chicken	C. jejuni	PRJNA591966	SRR10537890
18V3051F1	2018	Chicken	C. jejuni	PRJNA591966	SRR10537878
18V3055F1	2018	Chicken	C. jejuni	PRJNA591966	SRR10537873
18V3059F1	2018	Chicken	C. jejuni	PRJNA591966	SRR10537869
17Q3064F1	2017	Chicken	C. jejuni	PRJNA591966	SRR10537520
18A3008F1	2018	Chicken	C. jejuni	PRJNA591966	SRR10537778
18A3008F2	2018	Chicken	C. jejuni	PRJNA591966	SRR10537767
18A3020F1	2018	Chicken	C. jejuni	PRJNA591966	SRR10537612
18A3026F1	2018	Chicken	C. jejuni	PRJNA591966	SRR10537534
18A3061F1	2018	Chicken	C. jejuni	PRJNA591966	SRR10537885
18A3070F1	2018	Chicken	C. jejuni	PRJNA591966	SRR10537821
18N3071F1	2018	Lamb	C. jejuni	PRJNA591966	SRR10537663
18N3091F1	2018	Chicken	C. jejuni	PRJNA591966	SRR10537641
18V3052F1	2018	Chicken	C. jejuni	PRJNA591966	SRR10537877
19N3112F1	2019	Chicken	C. jejuni	PRJNA591966	SRR10537606
17N3020F1	2017	Chicken	C. jejuni	PRJNA591966	SRR10537811
17N3023F1	2017	Chicken	C. jejuni	PRJNA591966	SRR10537807
17N3043F1	2017	Lamb	C. jejuni	PRJNA591966	SRR10537793
17N3078F1	2017	Chicken	C. jejuni	PRJNA591966	SRR10537772
17N3250F1	2017	Chicken	C. jejuni	PRJNA591966	SRR10537725
17Q3054F1	2017	Chicken	C. jejuni	PRJNA591966	SRR10537531
17Q3117F1	2017	Chicken	C. jejuni	PRJNA591966	SRR10538072
17Q3118F1	2017	Chicken	C. jejuni	PRJNA591966	SRR10538071
17Q3123F1	2017	Chicken	C. jejuni	PRJNA591966	SRR10538066
17Q3141F1	2017	Chicken	C. jejuni	PRJNA591966	SRR10538048
18A3045F1	2018	Chicken	C. jejuni	PRJNA591966	SRR10538062
18A3053F1	2018	Beef	C. jejuni	PRJNA591966	SRR10537974
18A3066F1	2018	Chicken	C. jejuni	PRJNA591966	SRR10537830
18N3046F1	2018	Pork	C. jejuni	PRJNA591966	SRR10537685
18N3062F1	2018	Pork	C. jejuni	PRJNA591966	SRR10537672

18Q3013F1	2018	Chicken	C. jejuni	PRJNA591966	SRR10538034
18Q3025F1	2018	Chicken	C. jejuni	PRJNA591966	SRR10538022
19N3143F1	2019	Chicken	C. jejuni	PRJNA591966	SRR10537598

Table S2: Antimicrobial resistance genes and mutations used to infer phenotypic resistance in *Campylobacter* isolates.

**Drug class**	**Antimicrobial**	**Code**	**Gene**	**Mutation**	**Reference**
Aminoglycoside	Gentamicin	GEN	*aph(3')-IIIa*	None	Ramirez and Tolmasky (2010)
Beta-lactam	Ampicillin	AMP	*bla* $_{OXA-61}$	G57T	Zeng et al. (2014)
			bla $_{OXA-193}$<br>*bla* $_{OXA-184}$<br>*bla* $_{OXA-185}$	None	
Quinolone	Ciprofloxacin	CIP	*gyrA*	GyrA T86I	Hakanen et al. (2002)
Macrolide	Erythromycin	ERY	*erm* (B)	None	Qin et al. (2014)
			23S rRNA	A2074G, A2074C, A2074T, A2075G	Ladely et al. (2009)
Tetracycline	Tetracycline	TET	*tet* (O)	None	Whitehouse et al. (2018)

Table S3. Univariable results for hospitalisation, adjusted for age group, sex, and location, and final multivariable model
OR: odds ratio, aOR: adjusted odds ratio, CI: confidence interval, ref: reference category, Inf: no limit on confidence interval

			Univariable analysis		Final multivariable model	
**Variable**	**Hospitalised (N=115)**	**Not hospitalised (N=391)**	**OR (95% CI)**	**P value**	**aOR (95% CI)**	**P value**
Control variables						
Age group	115	391				
0-4yrs	4 (3.5%)	38 (9.7%)	0.4 (0.1-1.0)	0.072	0.3 (0.1-1.1)	0.089
5-14yrs	14 (12.2%)	44 (11.3%)	1.1 (0.5-2.2)	0.811	0.6 (0.3-1.5)	0.289
15-34yrs	30 (26.1%)	103 (26.3%)	ref		ref	
35-54yrs	21 (18.3%)	93 (23.8%)	0.8 (0.4-1.4)	0.424	0.9 (0.4-2.0)	0.826
55-74yrs	32 (27.8%)	93 (23.8%)	1.2 (0.7-2.1)	0.568	1.3 (0.7-2.7)	0.4
75+yrs	14 (12.2%)	20 (5.1%)	2.4 (1.1-5.3)	0.031	1.9 (0.7-4.9)	0.207
Gender	115	391				
Female	47 (40.9%)	169 (43.2%)	ref		ref	
Male	68 (59.1%)	222 (56.8%)	1.1 (0.7-1.7)	0.654	1.2 (0.7-2.1)	0.4
State	115	391				
ACT	31 (27.0%)	38 (9.7%)	ref		ref	
HNE (NSW)	68 (59.1%)	75 (19.2%)	1.1 (0.6-2.0)	0.72	1.2 (0.6-2.2)	0.609
Qld	16 (13.9%)	278 (71.1%)	0.1 (0.0-0.1)	< 0.001	0.1 (0.0-0.1)	< 0.001
Demographics and season						
Season	115	391				
Summer	32 (27.8%)	98 (25.1%)	ref			
Autumn	31 (27.0%)	99 (25.3%)	0.9 (0.5-1.8)	0.772		
Spring	23 (20.0%)	83 (21.2%)	0.7 (0.4-1.5)	0.391		
Winter	29 (25.2%)	111 (28.4%)	0.8 (0.4-1.5)	0.416		
Language other than English spoken at home	115	390				
No	104 (90.4%)	358 (91.8%)	ref			
Yes	11 (9.6%)	32 (8.2%)	0.8 (0.3-1.8)	0.591		
Aboriginal or Torres Strait Islander	115	390				
No	107 (93.0%)	373 (95.6%)	ref			
Yes	8 (7.0%)	17 (4.4%)	1.1 (0.4-2.9)	0.89		
Live in a rural or remote area	115	390				
No	104 (90.4%)	326 (83.6%)	ref			
Yes	11 (9.6%)	64 (16.4%)	0.6 (0.2-1.2)	0.155		
Highest education level of household	113	384				
Year 10	19 (16.8%)	40 (10.4%)	ref			
Year 12	14 (12.4%)	54 (14.1%)	0.7 (0.3-1.7)	0.412		
TAFE	30 (26.5%)	95 (24.7%)	1.0 (0.4-2.3)	0.932		
Undergraduate degree	33 (29.2%)	114 (29.7%)	1.1 (0.5-2.7)	0.762		
Postgraduate degree	17 (15.0%)	81 (21.1%)	0.6 (0.2-1.5)	0.245		

Variable	Hospitalised (N=115)	Not hospitalised (N=391)	Univariable analysis OR (95% CI)	P value	Final multivariable model aOR (95% CI)	P value
Total yearly household income	104	349				
<25k	14 (13.5%)	17 (4.9%)	ref			
25-50k	28 (26.9%)	53 (15.2%)	0.8 (0.3-2.2)	0.658		
50-100k	26 (25.0%)	107 (30.7%)	0.4 (0.2-1.2)	0.119		
100-150k	19 (18.3%)	83 (23.8%)	0.6 (0.2-1.7)	0.317		
>150k	17 (16.3%)	89 (25.5%)	0.4 (0.1-1.1)	0.086		
Illness symptoms						
Case experienced fever during illness	113	389				
No	25 (22.1%)	105 (27.0%)	ref			
Yes	88 (77.9%)	284 (73.0%)	1.1 (0.6-2.0)	0.807		
**Case experienced vomiting during illness**	115	391				
**No**	52 (45.2%)	266 (68.0%)	ref		ref	
**Yes**	63 (54.8%)	125 (32.0%)	2.6 (1.5-4.3)	< 0.001	**2.6 (1.5-4.6)**	**< 0.001**
**Case experienced stomach cramps during illness**	114	381				
**No**	22 (19.3%)	43 (11.3%)	ref		ref	
**Yes**	92 (80.7%)	338 (88.7%)	0.5 (0.2-1.0)	0.065	**0.4 (0.2-1.0)**	**0.045**
Case experienced blood in their stool during illness	109	372				
No	68 (62.4%)	222 (59.7%)	ref			
Yes	41 (37.6%)	150 (40.3%)	1.0 (0.6-1.7)	0.898		
Case experienced nausea during illness	114	371				
No	23 (20.2%)	113 (30.5%)	ref			
Yes	91 (79.8%)	258 (69.5%)	1.9 (1.0-3.5)	0.04		
Case experienced headaches during illness	110	356				
No	29 (26.4%)	126 (35.4%)	ref			
Yes	81 (73.6%)	230 (64.6%)	1.2 (0.6-2.2)	0.612		
Case experienced muscle and/or body aches during illness	110	364				
No	42 (38.2%)	110 (30.2%)	ref			
Yes	68 (61.8%)	254 (69.8%)	0.7 (0.4-1.2)	0.211		
Host health and medication factors						
Case took antibiotics in the four weeks prior to their illness	115	390				
No	109 (94.8%)	376 (96.4%)	ref			
Yes	6 (5.2%)	14 (3.6%)	1.0 (0.3-2.9)	0.951		
**Case took antibiotics as a result of their illness**	114	390				
**No**	48 (42.1%)	199 (51.0%)	ref		ref	
**Yes**	66 (57.9%)	191 (49.0%)	2.0 (1.2-3.3)	0.01	**1.8 (1.1-3.2)**	**0.026**

Variable	Hospitalised (N=115)	Not hospitalised (N=391)	Univariable analysis OR (95% CI)	Univariable analysis P value	Final multivariable model aOR (95% CI)	Final multivariable model P value
Taking regular medication to decrease stomach acid in 4 weeks prior to illness/interview	115	390				
No	82 (71.3%)	317 (81.3%)	ref			
Yes	33 (28.7%)	73 (18.7%)	1.8 (0.9-3.2)	0.073		
Used proton-pump inhibitors in 4 weeks prior to illness/interview	115	390				
No	86 (74.8%)	322 (82.6%)	ref			
Yes	29 (25.2%)	68 (17.4%)	1.6 (0.9-3.0)	0.135		
Used H2 receptor blockers in 4 weeks prior to illness/interview	115	390				
No	112 (97.4%)	386 (99.0%)	ref			
Yes	3 (2.6%)	4 (1.0%)	3.5 (0.5-21.8)	0.182		
**Chronic illness with diarrhoea or vomiting**	113	390				
**No**	97 (85.8%)	366 (93.8%)	ref		ref	
**Yes**	16 (14.2%)	24 (6.2%)	3.8 (1.6-9.2)	0.003	**4.4 (1.8-11.1)**	**0.001**
Took an immunosuppressive medication or had chemo- or radiation therapy in 4 weeks prior to illness	115	391				
No	106 (92.2%)	372 (95.1%)	ref			
Yes	9 (7.8%)	19 (4.9%)	0.9 (0.3-2.5)	0.906		
Had diarrhoeal illness for seven days or greater	114	389				
No	53 (46.5%)	205 (52.7%)	ref			
Yes	61 (53.5%)	184 (47.3%)	1.5 (0.9-2.5)	0.097		
<u>Pathogen factors</u>						
Campylobacter species	115	391				
C. jejuni	100 (87.0%)	322 (82.4%)	ref			
C. coli	15 (13.0%)	69 (17.6%)	0.6 (0.3-1.2)	0.153		
How many virulence genes were present in the isolate?	115	391				
1st quartile: ≤70 *C.coli;* ≤90 *C. jejuni*	30 (26.1%)	118 (30.2%)	ref			
2nd and 3rd quartiles: 70–≤73 *C. coli;* 90–≤101 *C. jejuni*	50 (43.5%)	187 (47.8%)	1.3 (0.7-2.4)	0.373		
4th quartile: >73 *C. coli; >101 C. jejuni*	35 (30.4%)	86 (22.0%)	1.3 (0.7-2.5)	0.466		
Ciprofloxacin resistance detected in isolate	115	390				
No	101 (87.8%)	342 (87.7%)	ref			
Yes	14 (12.2%)	48 (12.3%)	1.0 (0.5-2.1)	0.975		
Erythromycin resistance detected in isolate	115	391				
No	115 (100.0%)	390 (99.7%)	ref			
Yes	0 (0.0%)	1 (0.3%)	0.0 (Inf)	0.989		
Tetracycline resistance detected in isolate	115	391				
No	107 (93.0%)	350 (89.5%)	ref			
Yes	8 (7.0%)	41 (10.5%)	0.7 (0.3-1.6)	0.362		

Variable	Hospitalised (N=115)	Not hospitalised (N=391)	Univariable analysis OR (95% CI)	P value	Final multivariable model aOR (95% CI)	P value
Ampicillin resistance detected in isolate	113	372				
No	103 (91.2%)	357 (96.0%)	ref			
Yes	10 (8.8%)	15 (4.0%)	2.6 (0.9-7.0)	0.067		
Gentamicin resistance detected in isolate	115	391				
No	114 (99.1%)	390 (99.7%)	ref			
Yes	1 (0.9%)	1 (0.3%)	3.9 (0.1-158.3)	0.453		

Table S4. Univariable results for prescription of antibiotics following illness, adjusted for age group, sex, and location, and final multivariable model
OR: odds ratio, aOR: adjusted odds ratio, CI: confidence interval, ref: reference category, Inf: no limit on confidence interval

			**Univariable analysis**		**Final multivariable model**	
**Variable**	**Antibiotics prescribed (N=257)**	**No antibiotics prescribed (N=247)**	**OR (95% CI)**	**P value**	**aOR (95% CI)**	**P value**
Control variables						
Age group	257	247				
0-4yrs	16 (6.2%)	26 (10.5%)	0.5 (0.3-1.1)	0.079	0.5 (0.2-1.0)	0.067
5-14yrs	22 (8.6%)	36 (14.6%)	0.5 (0.3-1.0)	0.045	0.5 (0.3-1.1)	0.068
15-34yrs	71 (27.6%)	61 (24.7%)	ref		ref	
35-54yrs	66 (25.7%)	48 (19.4%)	1.2 (0.7-2.0)	0.518	1.3 (0.7-2.2)	0.417
55-74yrs	64 (24.9%)	60 (24.3%)	0.9 (0.6-1.5)	0.728	0.9 (0.5-1.5)	0.647
75+yrs	18 (7.0%)	16 (6.5%)	1.0 (0.5-2.1)	0.930	1.4 (0.6-3.2)	0.491
Gender	257	247				
Female	108 (42.0%)	108 (43.7%)	ref		ref	
Male	149 (58.0%)	139 (56.3%)	1.1 (0.8-1.5)	0.700	1.1 (0.8-1.6)	0.610
State	257	247				
ACT	34 (13.2%)	35 (14.2%)	ref		ref	
NSW	64 (24.9%)	77 (31.2%)	0.9 (0.5-1.5)	0.596	0.8 (0.5-1.6)	0.601
Qld	159 (61.9%)	135 (54.7%)	1.2 (0.7-2.1)	0.472	1.6 (0.9-2.9)	0.138
Demographics and season						
Season	257	247				
Summer	75 (29.2%)	53 (21.5%)	ref			
Autumn	69 (26.8%)	61 (24.7%)	0.8 (0.5-1.3)	0.342		
Spring	50 (19.5%)	56 (22.7%)	0.6 (0.4-1.1)	0.087		
Winter	63 (24.5%)	77 (31.2%)	0.6 (0.4-1.0)	0.033		
Language other than English spoken at home	256	247				
No	236 (92.2%)	224 (90.7%)	ref			
Yes	20 (7.8%)	23 (9.3%)	0.9 (0.5-1.8)	0.773		
Aboriginal or Torres Strait Islander	256	247				
No	243 (94.9%)	235 (95.1%)	ref			
Yes	13 (5.1%)	12 (4.9%)	1.3 (0.6-3.1)	0.527		
Live in a rural or remote area	256	247				
No	213 (83.2%)	216 (87.4%)	ref			
Yes	43 (16.8%)	31 (12.6%)	1.4 (0.8-2.4)	0.189		

Variable	Antibiotics prescribed (N=257)	No antibiotics prescribed (N=247)	Univariable analysis OR (95% CI)	P value	Final multivariable model aOR (95% CI)	P value
Highest education level of household	252	243				
Year 10	23 (9.1%)	36 (14.8%)	ref			
Year 12	39 (15.5%)	29 (11.9%)	2.2 (1.1-4.6)	0.038		
Technical and Further Education	71 (28.2%)	52 (21.4%)	2.4 (1.2-4.7)	0.012		
Undergraduate degree	70 (27.8%)	77 (31.7%)	1.5 (0.8-3.0)	0.206		
Postgraduate degree	49 (19.4%)	49 (20.2%)	1.8 (0.9-3.6)	0.106		
Total yearly household income	234	218				
<25k	15 (6.4%)	16 (7.3%)	ref			
25-50k	40 (17.1%)	41 (18.8%)	1.0 (0.4-2.4)	0.958		
50-100k	76 (32.5%)	57 (26.1%)	1.5 (0.6-3.4)	0.359		
100-150k	43 (18.4%)	58 (26.6%)	0.8 (0.3-2.0)	0.643		
>150k	60 (25.6%)	46 (21.1%)	1.4 (0.6-3.3)	0.46		
Illness symptoms						
Case experienced fever during illness	256	244				
No	66 (25.8%)	64 (26.2%)	ref			
Yes	190 (74.2%)	180 (73.8%)	1.1 (0.7-1.7)	0.664		
Case experienced vomiting during illness	257	247				
No	163 (63.4%)	154 (62.3%)	ref			
Yes	94 (36.6%)	93 (37.7%)	1.1 (0.7-1.6)	0.677		
Case experienced stomach cramps during illness	254	239				
No	32 (12.6%)	32 (13.4%)	ref			
Yes	222 (87.4%)	207 (86.6%)	1.2 (0.7-2.0)	0.599		
**Case experienced blood in their stool during illness**	247	234				
**No**	140 (56.7%)	150 (64.1%)	ref		ref	
**Yes**	107 (43.3%)	84 (35.9%)	1.6 (1.1-2.4)	0.019	**1.6 (1.1-2.4)**	**0.027**
Case experienced nausea during illness	250	233				
No	64 (25.6%)	72 (30.9%)	ref			
Yes	186 (74.4%)	161 (69.1%)	1.4 (0.9-2.1)	0.124		
Case experienced headaches during illness	242	222				
No	75 (31.0%)	80 (36.0%)	ref			
Yes	167 (69.0%)	142 (64.0%)	1.4 (0.9-2.1)	0.115		
Case experienced muscle and/or body aches during illness	247	226				
No	83 (33.6%)	69 (30.5%)	ref			
Yes	164 (66.4%)	157 (69.5%)	0.8 (0.5-1.2)	0.243		

Variable	Antibiotics prescribed (N=257)	No antibiotics prescribed (N=247)	Univariable analysis OR (95% CI)	P value	Final multivariable model aOR (95% CI)	P value
Host health and medication factors						
Case took antibiotics in the four weeks prior to their illness	257	246				
No	247 (96.1%)	236 (95.9%)	ref			
Yes	10 (3.9%)	10 (4.1%)	1.0 (0.4-2.6)	0.939		
Taking regular medication to decrease stomach acid in 4 weeks prior to illness/interview	257	246				
No	200 (77.8%)	198 (80.5%)	ref			
Yes	57 (22.2%)	48 (19.5%)	1.0 (0.6-1.6)	0.989		
Used proton-pump inhibitors in 4 weeks prior to illness/interview	257	246				
No	205 (79.8%)	201 (81.7%)	ref			
Yes	52 (20.2%)	45 (18.3%)	1.0 (0.6-1.5)	0.846		
Used H2 receptor blockers in 4 weeks prior to illness/interview	257	246				
No	252 (98.1%)	244 (99.2%)	ref			
Yes	5 (1.9%)	2 (0.8%)	2.0 (0.4-14.3)	0.411		
Chronic illness with diarrhoea or vomiting	255	246				
No	227 (89.0%)	234 (95.1%)	ref			
Yes	28 (11.0%)	12 (4.9%)	2.2 (1.1-4.5)	0.034		
Took an immunosuppressive medication or had chemo- or radiation therapy in 4 weeks prior to illness	257	247				
No	242 (94.2%)	235 (95.1%)	ref			
Yes	15 (5.8%)	12 (4.9%)	1.3 (0.6-2.9)	0.566		
**Hospitalised from illness**	257	247				
**No**	191 (74.3%)	199 (80.6%)	ref		ref	
**Yes**	66 (25.7%)	48 (19.4%)	1.9 (1.2-3.2)	0.009	**1.9 (1.1-3.2)**	**0.016**
Length of hospital stay	256	246				
0 days	191 (74.6%)	199 (80.9%)	ref			
1 day	11 (4.3%)	16 (6.5%)	1.0 (0.4-2.2)	0.924		
2-4 days	40 (15.6%)	25 (10.2%)	2.4 (1.3-4.5)	0.005		
5-7 days	8 (3.1%)	6 (2.4%)	1.8 (0.6-5.9)	0.301		
8-10 days	6 (2.3%)	0 (0.0%)	N/A	0.979		
**Had diarrhoeal illness for seven days or greater**	256	245				
**No**	110 (43.0%)	147 (60.0%)	ref		ref	
**Yes**	146 (57.0%)	98 (40.0%)	2.0 (1.4-2.9)	< 0.001	**2.0 (1.3-2.9)**	**0.001**

Variable	Antibiotics prescribed (N=257)	No antibiotics prescribed (N=247)	Univariable analysis OR (95% CI)	Univariable analysis P value	Final multivariable model aOR (95% CI)	Final multivariable model P value
Pathogen factors						
Campylobacter species	257	247				
C. jejuni	214 (83.3%)	206 (83.4%)	ref			
C. coli	43 (16.7%)	41 (16.6%)	0.9 (0.6-1.5)	0.703		
How many virulence genes were present in the isolate?	257	247				
1st quartile: ≤70 *C.coli;* ≤ 90 *C. jejuni*	74 (28.8%)	73 (29.6%)	ref			
2nd and 3rd quartiles: 70–≤73 *C. coli;* 90–≤101 *C. jejuni*	117 (45.5%)	120 (48.6%)	1.0 (0.7-1.5)	0.982		
4th quartile: >73 *C. coli; >101 C. jejuni*	66 (25.7%)	54 (21.9%)	1.3 (0.8-2.1)	0.311		
Ciprofloxacin resistance detected in isolate	256	247				
No	229 (89.5%)	212 (85.8%)	ref			
Yes	27 (10.5%)	35 (14.2%)	0.7 (0.4-1.2)	0.166		
Erythromycin resistance detected in isolate	257	247				
No	256 (99.6%)	247 (100.0%)	ref			
Yes	1 (0.4%)	0 (0.0%)	N/A	0.98		
Tetracycline resistance detected in isolate	257	247				
No	229 (89.1%)	227 (91.9%)	ref			
Yes	28 (10.9%)	20 (8.1%)	1.3 (0.7-2.4)	0.39		
Ampicillin resistance detected in isolate	248	235				
No	237 (95.6%)	221 (94.0%)	ref			
Yes	11 (4.4%)	14 (6.0%)	0.8 (0.3-1.9)	0.61		
Gentamicin resistance detected in isolate	257	247				
No	255 (99.2%)	247 (100.0%)	ref			
Yes	2 (0.8%)	0 (0.0%)	N/A	0.981		

Table S5. Univariable results for length of diarrhoeal illness, adjusted for age group, sex, and location, and final multivariable model
OR: odds ratio, aOR: adjusted odds ratio, CI: confidence interval, ref: reference category, Inf: no limit on confidence interval

			Univariable analysis		Final multivariable model	
**Variable**	**< 7 days (N=258)**	**7 days + (N=245)**	**OR (95% CI)**	**P value**	**aOR (95% CI)**	**P value**
Control variables						
Age group	258	245				
0-4yrs	22 (8.5%)	20 (8.2%)	0.4 (0.1-1.0)	0.072	1.0 (0.3-3.1)	0.947
5-14yrs	34 (13.2%)	24 (9.8%)	1.1 (0.5-2.2)	0.811	1.0 (0.5-2.0)	0.972
15-34yrs	76 (29.5%)	56 (22.9%)	ref		ref	
35-54yrs	60 (23.3%)	53 (21.6%)	0.8 (0.4-1.4)	0.424	1.0 (0.6-1.8)	0.903
55-74yrs	49 (19.0%)	76 (31.0%)	1.2 (0.7-2.1)	0.568	1.9 (1.1-3.3)	0.013
75+yrs	17 (6.6%)	16 (6.5%)	2.4 (1.1-5.3)	0.031	1.3 (0.6-3.1)	0.484
Gender	258	245				
Female	108 (41.9%)	107 (43.7%)	ref		ref	
Male	150 (58.1%)	138 (56.3%)	1.1 (0.7-1.7)	0.654	1.0 (0.7-1.5)	0.892
State	258	245				
ACT	39 (15.1%)	29 (11.8%)	ref		ref	
NSW	74 (28.7%)	69 (28.2%)	1.1 (0.6-2.0)	0.720	1.1 (0.6-2.1)	0.660
Qld	145 (56.2%)	147 (60.0%)	0.1 (0.0-0.1)	< 0.001	1.4 (0.8-2.5)	0.261
Demographics and season						
Season	258	245				
Summer	63 (24.4%)	67 (27.3%)	ref			
Autumn	60 (23.3%)	68 (27.8%)	1.1 (0.7-1.8)	0.731		
Spring	58 (22.5%)	48 (19.6%)	0.8 (0.5-1.3)	0.334		
Winter	77 (29.8%)	62 (25.3%)	0.8 (0.5-1.3)	0.428		
Language other than English spoken at home	257	245				
No	227 (88.3%)	232 (94.7%)	ref			
Yes	30 (11.7%)	13 (5.3%)	0.5 (0.2-0.9)	0.035		
Aboriginal or Torres Strait Islander	257	245				
No	245 (95.3%)	232 (94.7%)	ref			
Yes	12 (4.7%)	13 (5.3%)	1.2 (0.5-2.9)	0.611		
Live in a rural or remote area	257	245				
No	225 (87.5%)	202 (82.4%)	ref			
Yes	32 (12.5%)	43 (17.6%)	1.5 (0.9-2.5)	0.142		

Variable	< 7 days (N=258)	7 days + (N=245)	Univariable analysis OR (95% CI)	P value	Final multivariable model aOR (95% CI)	P value
Highest education level of household	252	242				
Year 10	29 (11.5%)	30 (12.4%)	ref			
Year 12	27 (10.7%)	39 (16.1%)	1.7 (0.8-3.6)	0.163		
Technical and Further Education	70 (27.8%)	55 (22.7%)	0.9 (0.5-1.8)	0.874		
Undergraduate degree	76 (30.2%)	70 (28.9%)	1.1 (0.6-2.1)	0.752		
Postgraduate degree	50 (19.8%)	48 (19.8%)	1.2 (0.6-2.5)	0.547		
Total yearly household income	236	214				
<25k	14 (5.9%)	17 (7.9%)	ref			
25-50k	44 (18.6%)	36 (16.8%)	0.7 (0.3-1.7)	0.490		
50-100k	66 (28.0%)	66 (30.8%)	1.1 (0.5-2.4)	0.899		
100-150k	53 (22.5%)	49 (22.9%)	1.0 (0.4-2.4)	0.954		
>150k	59 (25.0%)	46 (21.5%)	0.9 (0.4-2.2)	0.823		
Illness symptoms						
Case experienced fever during illness	255	244				
No	65 (25.5%)	64 (26.2%)	ref			
Yes	190 (74.5%)	180 (73.8%)	1.1 (0.7-1.7)	0.689		
Case experienced vomiting during illness	258	245				
No	168 (65.1%)	149 (60.8%)	ref			
Yes	90 (34.9%)	96 (39.2%)	1.3 (0.9-2.0)	0.132		
Case experienced stomach cramps during illness	252	240				
No	30 (11.9%)	34 (14.2%)	ref			
Yes	222 (88.1%)	206 (85.8%)	1.0 (0.6-1.7)	0.960		
Case experienced blood in their stool during illness	245	233				
No	149 (60.8%)	138 (59.2%)	ref			
Yes	96 (39.2%)	95 (40.8%)	1.3 (0.9-1.9)	0.234		
Case experienced nausea during illness	247	235				
No	74 (30.0%)	61 (26.0%)	ref			
Yes	173 (70.0%)	174 (74.0%)	1.3 (0.9-2.0)	0.174		
**Case experienced headaches during illness**	236	227				
**No**	88 (37.3%)	65 (28.6%)	ref		ref	
**Yes**	148 (62.7%)	162 (71.4%)	1.7 (1.1-2.6)	0.017	**1.7 (1.1-2.6)**	**0.021**

Variable	< 7 days (N=258)	7 days + (N=245)	Univariable analysis OR (95% CI)	P value	Final multivariable model aOR (95% CI)	P value
Case experienced muscle and/or body aches during illness	240	231				
No	79 (32.9%)	72 (31.2%)	ref			
Yes	161 (67.1%)	159 (68.8%)	1.2 (0.8-1.8)	0.414		
Host health and medication factors						
Case took antibiotics in the four weeks prior to their illness	258	244				
No	248 (96.1%)	234 (95.9%)	ref			
Yes	10 (3.9%)	10 (4.1%)	1.0 (0.4-2.5)	0.989		
**Case took antibiotics as a result of their illness**	257	244				
**No**	147 (57.2%)	98 (40.2%)	ref		ref	
**Yes**	110 (42.8%)	146 (59.8%)	2.0 (1.4-2.9)	< 0.001	**2.0 (1.4-3.0)**	**0.000**
Taking regular medication to decrease stomach acid in 4 weeks prior to illness/interview	258	244				
No	219 (84.9%)	179 (73.4%)	ref			
Yes	39 (15.1%)	65 (26.6%)	1.9 (1.2-3.0)	0.012		
**Used proton-pump inhibitors in 4 weeks prior to illness/interview**	258	244				
**No**	222 (86.0%)	185 (75.8%)	ref		ref	
**Yes**	36 (14.0%)	59 (24.2%)	1.8 (1.1-3.0)	0.018	**2.0 (1.2-3.3)**	**0.010**
Used H2 receptor blockers in 4 weeks prior to illness/interview	258	244				
No	256 (99.2%)	239 (98.0%)	ref			
Yes	2 (0.8%)	5 (2.0%)	2.8 (0.6-19.9)	0.237		
Chronic illness with diarrhoea or vomiting	257	243				
No	240 (93.4%)	220 (90.5%)	ref			
Yes	17 (6.6%)	23 (9.5%)	1.4 (0.7-2.8)	0.321		
Took an immunosuppressive medication or had chemo- or radiation therapy in 4 weeks prior to illness	258	245				
No	245 (95.0%)	230 (93.9%)	ref			
Yes	13 (5.0%)	15 (6.1%)	1.1 (0.5-2.5)	0.819		
Hospitalised from illness	258	245				
No	205 (79.5%)	184 (75.1%)	ref			
Yes	53 (20.5%)	61 (24.9%)	1.5 (0.9-2.5)	0.101		

Variable	< 7 days (N=258)	7 days + (N=245)	Univariable analysis OR (95% CI)	P value	Final multivariable model aOR (95% CI)	P value
Length of hospital stay	257	245				
0 days	205 (79.8%)	184 (75.1%)	ref			
1 day	14 (5.4%)	14 (5.7%)	1.4 (0.6-3.1)	0.450		
2-4 days	35 (13.6%)	30 (12.2%)	1.1 (0.6-2.0)	0.737		
5-7 days	3 (1.2%)	11 (4.5%)	4.7 (1.4-21.8)	0.024		
8-10 days	0 (0.0%)	6 (2.4%)	N/A	0.978		
<u>Pathogen factors</u>						
Campylobacter species	258	245				
C. jejuni	221 (85.7%)	199 (81.2%)	ref			
C. coli	37 (14.3%)	46 (18.8%)	1.3 (0.8-2.2)	0.238		
How many virulence genes were present in the isolate?	258	245				
1st quartile: ≤70 *C.coli;* ≤90 *C. jejuni*	77 (29.8%)	70 (28.6%)	ref			
2nd and 3rd quartiles: 70–≤73 *C. coli;* 90–≤101 *C. jejuni*	118 (45.7%)	119 (48.6%)	1.2 (0.8-1.8)	0.465		
4th quartile: >73 *C. coli; >101 C. jejuni*	63 (24.4%)	56 (22.9%)	1.1 (0.6-1.7)	0.829		
Ciprofloxacin resistance detected in isolate	257	245				
No	227 (88.3%)	213 (86.9%)	ref			
Yes	30 (11.7%)	32 (13.1%)	1.1 (0.7-2.0)	0.635		
Erythromycin resistance detected in isolate	258	245				
No	258 (100.0%)	244 (99.6%)	ref			
Yes	0 (0.0%)	1 (0.4%)	N/A	0.980		
Tetracycline resistance detected in isolate	258	245				
No	229 (88.8%)	226 (92.2%)	ref			
Yes	29 (11.2%)	19 (7.8%)	0.7 (0.4-1.2)	0.198		
Ampicillin resistance detected in isolate	249	234				
No	234 (94.0%)	224 (95.7%)	ref			
Yes	15 (6.0%)	10 (4.3%)	0.7 (0.3-1.6)	0.377		
Gentamicin resistance detected in isolate	258	245				
No	258 (100.0%)	243 (99.2%)	ref			
Yes	0 (0.0%)	2 (0.8%)	N/A	0.981		

S6 Table. Summary of multi-locus sequence type (MLST) and virulence gene prevalence in *Campylobacter jejuni* and *C. coli* human isolates.

**Jejuni MLST (n)**	Median virulence genes present (range)	**Coli MLST (n)**	Median virulence genes present (range)
10152 (n=1)	81 (81 - 81)	1243 (n=1)	63 (63 - 63)
538 (n=7)	83 (82 - 83)	10173 (n=1)	64 (64 - 64)
10139 (n=1)	83 (83 - 83)	10168 (n=1)	67 (67 - 67)
257 (n=10)	84 (84 - 84)	825 (n=12)	70 (69 - 70)
583 (n=5)	84 (84 - 84)	829 (n=2)	70 (70 - 70)
137 (n=4)	84 (84 - 88)	1181 (n=23)	71 (68 - 73)
10127 (n=2)	84 (84 - 84)	9436 (n=10)	71 (70 - 72)
692 (n=1)	84 (84 - 84)	4175 (n=3)	72 (72 - 74)
2083 (n=18)	85 (84 - 86)	831 (n=2)	72 (71 - 72)
534 (n=4)	85 (85 - 85)	9420 (n=2)	72 (71 - 72)
10131 (n=1)	86 (86 - 86)	860 (n=7)	73 (71 - 76)
567 (n=20)	87 (87 - 87)	2179 (n=1)	73 (73 - 73)
128 (n=3)	87 (87 - 88)	827 (n=9)	74 (69 - 77)
10146 (n=1)	87 (87 - 87)	9419 (n=3)	74 (72 - 76)
354 (n=1)	87 (87 - 87)	10158 (n=1)	74 (74 - 74)
449 (n=2)	88 (87 - 88)	10172 (n=1)	75 (75 - 75)
9173 (n=1)	88 (88 - 88)	8926 (n=1)	75 (75 - 75)
9592 (n=1)	88 (88 - 88)	1055 (n=1)	76 (76 - 76)
528 (n=14)	89 (88 - 90)	9435 (n=1)	76 (76 - 76)
51 (n=4)	89 (89 - 90)	6110 (n=1)	82 (82 - 82)
9608 (n=1)	89 (89 - 89)	10157 (n=1)	85 (85 - 85)
2398 (n=15)	90 (89 - 90)		
1525 (n=1)	90 (90 - 90)		
4577 (n=1)	90 (90 - 90)		
42 (n=11)	91 (90 - 92)		
45 (n=11)	91 (89 - 94)		
8436 (n=2)	91 (91 - 91)		
61 (n=13)	92 (91 - 97)		
49 (n=4)	92 (91 - 92)		
4080 (n=1)	92 (92 - 92)		
6788 (n=1)	92 (92 - 92)		
820 (n=1)	92 (92 - 92)		
52 (n=7)	93 (93 - 94)		
10143 (n=3)	93 (88 - 95)		
658 (n=13)	94 (85 - 95)		
520 (n=5)	94 (93 - 95)		
7323 (n=5)	94 (94 - 95)		
535 (n=2)	94 (94 - 94)		
933 (n=2)	94 (94 - 95)		
9425 (n=1)	94 (94 - 94)		
48 (n=21)	95 (93 - 97)		
508 (n=10)	95 (95 - 95)		
2343 (n=7)	95 (94 - 96)		
10130 (n=5)	95 (94 - 96)		
4684 (n=1)	95 (95 - 95)		
38 (n=2)	96 (96 - 97)		
161 (n=1)	97 (97 - 97)		
1911 (n=1)	97 (97 - 97)		
696 (n=6)	98 (96 - 98)		
50 (n=75)	99 (94 - 104)		
4896 (n=9)	101 (98 - 102)		
46 (n=24)	102 (95 - 104)		
10138 (n=1)	102 (102 - 102)		
227 (n=1)	102 (102 - 102)		
1728 (n=1)	103 (103 - 103)		
2947 (n=1)	105 (105 - 105)		
6891 (n=1)	105 (105 - 105)		
525 (n=10)	106 (105 - 107)		
9432 (n=5)	108 (107 - 109)		
53 (n=9)	114 (97 - 115)		
982 (n=1)	114 (114 - 114)		
21 (n=13)	115 (111 - 117)		
10132 (n=8)	116 (114 - 118)		
190 (n=4)	116 (116 - 116)		
5687 (n=1)	116 (116 - 116)		

Table S7. Comparison of isolate virulence gene prevalence between human and retail meat isolates, Australia, 2018–2019.
^Pearson's Chi-squared test with Yates' continuity correction. N/A: no gene present, or no analysis possible due to zero levels.
* indicates significant result (p < 0.05).

	Campylobacter jejuni				***Campylobacter coli***		
	Food (*n* =285)	Human (*n* =422)	Chi-square		Food (*n* =331)	Human (*n* =84)	Chi-square
Gene	Count (%)	Count (%)	*p* -value^	Gene	Count (%)	Count (%)	*p* -value^
cadF			1.000	***cadF***			1.000
Yes	285 (100.0%)	422 (100.0%)		No	1 (0.3%)	0 (0.0%)	
				Yes	330 (99.7%)	84 (100.0%)	
Cj1416c			0.146	***Cj1416c***			1.000
No	50 (17.5%)	56 (13.3%)		No	329 (99.4%)	83 (98.8%)	
Yes	235 (82.5%)	366 (86.7%)		Yes	2 (0.6%)	1 (1.2%)	
Cj1417c			0.115	***Cj1417c***			0.250
No	51 (17.9%)	56 (13.3%)		No	310 (93.7%)	82 (97.6%)	
Yes	234 (82.1%)	366 (86.7%)		Yes	21 (6.3%)	2 (2.4%)	
Cj1419c			0.107	***Cj1419c***			0.287
No	49 (17.2%)	53 (12.6%)		No	311 (94.0%)	82 (97.6%)	
Yes	236 (82.8%)	369 (87.4%)		Yes	20 (6.0%)	2 (2.4%)	
Cj1420c			0.398	***Cj1420c***			0.287
No	48 (16.8%)	60 (14.2%)		No	311 (94.0%)	82 (97.6%)	
Yes	237 (83.2%)	362 (85.8%)		Yes	20 (6.0%)	2 (2.4%)	
Cj1421c			0.000 *	***Cj1421c***			N/A
No	277 (97.2%)	374 (88.6%)		No	331 (100.0%)	84 (100.0%)	
Yes	8 (2.8%)	48 (11.4%)					
Cj1422c			0.000 *	***Cj1422c***			0.867
No	282 (98.9%)	379 (89.8%)		No	330 (99.7%)	83 (98.8%)	
Yes	3 (1.1%)	43 (10.2%)		Yes	1 (0.3%)	1 (1.2%)	
Cj1426c			0.782	***Cj1426c***			N/A
No	254 (89.1%)	372 (88.2%)		No	331 (100.0%)	84 (100.0%)	
Yes	31 (10.9%)	50 (11.8%)					
Cj1427c			0.105	***Cj1427c***			0.867
No	204 (71.6%)	326 (77.3%)		No	330 (99.7%)	83 (98.8%)	
Yes	81 (28.4%)	96 (22.7%)		Yes	1 (0.3%)	1 (1.2%)	
Cj1432c			0.634	***Cj1432c***			0.867
No	256 (89.8%)	373 (88.4%)		No	330 (99.7%)	83 (98.8%)	
Yes	29 (10.2%)	49 (11.6%)		Yes	1 (0.3%)	1 (1.2%)	
Cj1434c			0.128	***Cj1434c***			1.000
No	282 (98.9%)	409 (96.9%)		No	330 (99.7%)	84 (100.0%)	
Yes	3 (1.1%)	13 (3.1%)		Yes	1 (0.3%)	0 (0.0%)	
Cj1435c			0.812	***Cj1435c***			0.867
No	248 (87.0%)	371 (87.9%)		No	330 (99.7%)	83 (98.8%)	
Yes	37 (13.0%)	51 (12.1%)		Yes	1 (0.3%)	1 (1.2%)	
Cj1436c			0.812	***Cj1436c***			0.867
No	248 (87.0%)	371 (87.9%)		No	330 (99.7%)	83 (98.8%)	
Yes	37 (13.0%)	51 (12.1%)		Yes	1 (0.3%)	1 (1.2%)	
Cj1437c			0.920	***Cj1437c***			N/A
No	249 (87.4%)	371 (87.9%)		No	331 (100.0%)	84 (100.0%)	
Yes	36 (12.6%)	51 (12.1%)					
Cj1438c			0.137	***Cj1438c***			N/A
No	279 (97.9%)	403 (95.5%)		No	331 (100.0%)	84 (100.0%)	
Yes	6 (2.1%)	19 (4.5%)					
Cj1440c			0.604	***Cj1440c***			0.867
No	255 (89.5%)	371 (87.9%)		No	330 (99.7%)	83 (98.8%)	
Yes	30 (10.5%)	51 (12.1%)		Yes	1 (0.3%)	1 (1.2%)	
cysC			0.088	***cysC***			1.000
No	76 (26.7%)	88 (20.9%)		No	329 (99.4%)	83 (98.8%)	
Yes	209 (73.3%)	334 (79.1%)		Yes	2 (0.6%)	1 (1.2%)	
fcl			0.609	***fcl***			1.000
No	242 (84.9%)	351 (83.2%)		No	329 (99.4%)	84 (100.0%)	
Yes	43 (15.1%)	71 (16.8%)		Yes	2 (0.6%)	0 (0.0%)	

	Campylobacter jejuni				*Campylobacter coli*		
	Food (*n* =285)	Human (*n* =422)	Chi-square		Food (*n* =331)	Human (*n* =84)	Chi-square
Gene	Count (%)	Count (%)	*p* -value^	Gene	Count (%)	Count (%)	*p* -value^
glf			0.812	***glf***			0.867
No	248 (87.0%)	371 (87.9%)		No	330 (99.7%)	83 (98.8%)	
Yes	37 (13.0%)	51 (12.1%)		Yes	1 (0.3%)	1 (1.2%)	
hddA			1.000	***hddA***			0.174
No	74 (26.0%)	109 (25.8%)		No	216 (65.3%)	62 (73.8%)	
Yes	211 (74.0%)	313 (74.2%)		Yes	115 (34.7%)	22 (26.2%)	
hddC			0.959	***hddC***			0.585
No	77 (31.2%)	116 (31.3%)		No	328 (99.1%)	82 (97.6%)	
Yes	170 (68.8%)	255 (68.7%)		Yes	3 (0.9%)	2 (2.4%)	
kfiD			0.956	***kfiD***			0.867
No	250 (87.7%)	372 (88.2%)		No	330 (99.7%)	83 (98.8%)	
Yes	35 (12.3%)	50 (11.8%)		Yes	1 (0.3%)	1 (1.2%)	
kpsC			0.044 *	***kpsC***			1.000
No	26 (9.1%)	21 (5.0%)		No	329 (99.4%)	83 (98.8%)	
Yes	259 (90.9%)	401 (95.0%)		Yes	2 (0.6%)	1 (1.2%)	
kpsD			1.000	***kpsD***			1.000
Yes	285 (100.0%)	422 (100.0%)		No	2 (0.6%)	1 (1.2%)	
				Yes	329 (99.4%)	83 (98.8%)	
kpsE			1.000	***kpsE***			0.585
No	1 (0.4%)	1 (0.2%)		No	328 (99.1%)	82 (97.6%)	
Yes	284 (99.6%)	421 (99.8%)		Yes	3 (0.9%)	2 (2.4%)	
kpsF			0.658	***kpsF***			1.000
No	0 (0.0%)	2 (0.5%)		Yes	331 (100.0%)	84 (100.0%)	
Yes	285 (100.0%)	418 (99.5%)					
kpsM			0.843	***kpsM***			1.000
No	1 (0.4%)	0 (0.0%)		No	257 (77.6%)	65 (77.4%)	
Yes	284 (99.6%)	422 (100.0%)		Yes	74 (22.4%)	19 (22.6%)	
kpsS			1.000	***kpsS***			1.000
Yes	285 (100.0%)	422 (100.0%)		No	1 (0.3%)	0 (0.0%)	
				Yes	330 (99.7%)	84 (100.0%)	
kpsT			1.000	***kpsT***			1.000
Yes	285 (100.0%)	421 (100.0%)		No	1 (0.3%)	0 (0.0%)	
				Yes	330 (99.7%)	84 (100.0%)	
rfbC			0.022 *	***rfbC***			0.413
No	137 (48.1%)	165 (39.1%)		No	239 (72.2%)	65 (77.4%)	
Yes	148 (51.9%)	257 (60.9%)		Yes	92 (27.8%)	19 (22.6%)	
cdtA			0.355	***cdtA***			1.000
No	5 (1.8%)	3 (0.7%)		No	329 (99.4%)	84 (100.0%)	
Yes	280 (98.2%)	419 (99.3%)		Yes	2 (0.6%)	0 (0.0%)	
cdtB			0.658	***cdtB***			N/A
No	0 (0.0%)	2 (0.5%)		No	331 (100.0%)	84 (100.0%)	
Yes	285 (100.0%)	420 (99.5%)					
cdtC			1.000	***cdtC***			N/A
Yes	285 (100.0%)	422 (100.0%)		No	331 (100.0%)	84 (100.0%)	
ciaB			0.358	***ciaB***			0.603
No	11 (3.9%)	10 (2.4%)		No	19 (5.7%)	3 (3.6%)	
Yes	274 (96.1%)	412 (97.6%)		Yes	312 (94.3%)	81 (96.4%)	
ciaC			1.000	***ciaC***			1.000
Yes	285 (100.0%)	422 (100.0%)		Yes	331 (100.0%)	84 (100.0%)	
cheA			0.366	***cheA***			0.699
No	4 (1.4%)	2 (0.5%)		No	4 (1.2%)	0 (0.0%)	
Yes	281 (98.6%)	420 (99.5%)		Yes	327 (98.8%)	84 (100.0%)	
cheV			0.054 *	***cheV***			1.000
No	4 (1.4%)	0 (0.0%)		No	2 (0.6%)	0 (0.0%)	
Yes	281 (98.6%)	422 (100.0%)		Yes	329 (99.4%)	84 (100.0%)	

	Campylobacter jejuni		
Gene	Food (n =285) Count (%)	Human (n =422) Count (%)	Chi-square p -value^
cheW			0.317
No	2 (0.7%)	0 (0.0%)	
Yes	283 (99.3%)	422 (100.0%)	
cheY			1.000
Yes	285 (100.0%)	422 (100.0%)	
eptC			1.000
Yes	285 (100.0%)	422 (100.0%)	
flaA			1.000
Yes	285 (100.0%)	422 (100.0%)	
flaB			1.000
Yes	285 (100.0%)	422 (100.0%)	
flaC			1.000
Yes	285 (100.0%)	421 (100.0%)	
flaD			1.000
Yes	285 (100.0%)	422 (100.0%)	
flaG			1.000
Yes	285 (100.0%)	422 (100.0%)	
flgA			1.000
No	0 (0.0%)	1 (0.2%)	
Yes	285 (100.0%)	421 (99.8%)	
flgB			1.000
Yes	285 (100.0%)	422 (100.0%)	
flgC			1.000
Yes	285 (100.0%)	422 (100.0%)	
flgD			1.000
Yes	285 (100.0%)	422 (100.0%)	
flgE			1.000
Yes	192 (100.0%)	278 (100.0%)	
flgF			1.000
Yes	285 (100.0%)	421 (100.0%)	
flgG			1.000
Yes	285 (100.0%)	421 (100.0%)	
flgH			1.000
Yes	285 (100.0%)	421 (100.0%)	
flgI			1.000
Yes	285 (100.0%)	422 (100.0%)	
flgJ			0.843
No	1 (0.4%)	0 (0.0%)	
Yes	284 (99.6%)	422 (100.0%)	
flgK			1.000
Yes	285 (100.0%)	422 (100.0%)	
flgM			1.000
Yes	285 (100.0%)	422 (100.0%)	
flgP			1.000
Yes	285 (100.0%)	422 (100.0%)	
flgQ			1.000
Yes	285 (100.0%)	422 (100.0%)	

	Campylobacter coli		
Gene	Food (n =331) Count (%)	Human (n =84) Count (%)	Chi-square p -value^
cheW			1.000
Yes	331 (100.0%)	84 (100.0%)	
cheY			1.000
Yes	331 (100.0%)	84 (100.0%)	
eptC			1.000
No	313 (94.6%)	79 (94.0%)	
Yes	18 (5.4%)	5 (6.0%)	
flaA			1.000
Yes	331 (100.0%)	84 (100.0%)	
flaB			1.000
Yes	331 (100.0%)	84 (100.0%)	
flaC			1.000
Yes	331 (100.0%)	84 (100.0%)	
flaD			1.000
No	1 (0.3%)	0 (0.0%)	
Yes	330 (99.7%)	84 (100.0%)	
flaG			0.787
No	8 (2.4%)	1 (1.2%)	
Yes	323 (97.6%)	83 (98.8%)	
flgA			0.070
No	314 (94.9%)	84 (100.0%)	
Yes	17 (5.1%)	0 (0.0%)	
flgB			1.000
Yes	331 (100.0%)	84 (100.0%)	
flgC			1.000
Yes	331 (100.0%)	84 (100.0%)	
flgD			0.916
No	7 (2.1%)	1 (1.2%)	
Yes	324 (97.9%)	83 (98.8%)	
flgE			1.000
No	1 (0.3%)	0 (0.0%)	
Yes	330 (99.7%)	84 (100.0%)	
flgF			1.000
Yes	331 (100.0%)	84 (100.0%)	
flgG			1.000
Yes	331 (100.0%)	84 (100.0%)	
flgH			1.000
No	1 (0.3%)	0 (0.0%)	
Yes	330 (99.7%)	84 (100.0%)	
flgI			1.000
Yes	331 (100.0%)	84 (100.0%)	
flgJ			1.000
No	1 (0.3%)	0 (0.0%)	
Yes	330 (99.7%)	84 (100.0%)	
flgK			1.000
No	1 (0.3%)	0 (0.0%)	
Yes	330 (99.7%)	84 (100.0%)	
flgM			1.000
No	1 (0.3%)	0 (0.0%)	
Yes	330 (99.7%)	84 (100.0%)	
flgP			1.000
Yes	331 (100.0%)	84 (100.0%)	
flgQ			0.986
No	21 (6.3%)	6 (7.1%)	
Yes	310 (93.7%)	78 (92.9%)	

	Campylobacter jejuni			
	Food (n =285)	Human (n =422)	Chi-square	
Gene	Count (%)	Count (%)	p -value^	
flgR			1.000	
Yes	285 (100.0%)	422 (100.0%)		
flgS			1.000	
No	0 (0.0%)	1 (0.2%)		
Yes	285 (100.0%)	421 (99.8%)		
flhA			1.000	
No	0 (0.0%)	1 (0.2%)		
Yes	285 (100.0%)	421 (99.8%)		
flhB			0.843	
No	1 (0.4%)	0 (0.0%)		
Yes	284 (99.6%)	422 (100.0%)		
flhF			1.000	
Yes	285 (100.0%)	422 (100.0%)		
flhG			1.000	
Yes	285 (100.0%)	422 (100.0%)		
fliA			1.000	
Yes	285 (100.0%)	422 (100.0%)		
fliD			0.443	
No	1 (0.4%)	5 (1.2%)		
Yes	284 (99.6%)	417 (98.8%)		
fliE			1.000	
Yes	285 (100.0%)	421 (100.0%)		
fliF			1.000	
Yes	285 (100.0%)	422 (100.0%)		
fliG			1.000	
Yes	285 (100.0%)	422 (100.0%)		
fliH			1.000	
Yes	285 (100.0%)	422 (100.0%)		
fliI			1.000	
Yes	285 (100.0%)	422 (100.0%)		
fliK			0.000	*
No	58 (20.4%)	15 (3.6%)		
Yes	227 (79.6%)	407 (96.4%)		
fliL			1.000	
Yes	285 (100.0%)	422 (100.0%)		
fliM			1.000	
Yes	285 (100.0%)	422 (100.0%)		
fliN			1.000	
Yes	285 (100.0%)	421 (100.0%)		
fliP			1.000	
Yes	285 (100.0%)	422 (100.0%)		
fliQ			1.000	
Yes	285 (100.0%)	420 (100.0%)		
fliR			1.000	
Yes	285 (100.0%)	422 (100.0%)		
fliS			1.000	
Yes	285 (100.0%)	422 (100.0%)		
fliW			1.000	
Yes	285 (100.0%)	422 (100.0%)		

	Campylobacter coli		
	Food (n =331)	Human (n =84)	Chi-square
Gene	Count (%)	Count (%)	p -value^
flgR			1.000
No	3 (0.9%)	1 (1.2%)	
Yes	328 (99.1%)	83 (98.8%)	
flgS			1.000
Yes	331 (100.0%)	84 (100.0%)	
flhA			1.000
Yes	331 (100.0%)	84 (100.0%)	
flhB			1.000
Yes	331 (100.0%)	84 (100.0%)	
flhF			1.000
No	1 (0.3%)	0 (0.0%)	
Yes	330 (99.7%)	84 (100.0%)	
flhG			1.000
Yes	331 (100.0%)	84 (100.0%)	
fliA			1.000
Yes	331 (100.0%)	84 (100.0%)	
fliD			0.087
No	34 (10.3%)	3 (3.6%)	
Yes	297 (89.7%)	81 (96.4%)	
fliE			1.000
Yes	331 (100.0%)	84 (100.0%)	
fliF			1.000
Yes	331 (100.0%)	84 (100.0%)	
fliG			1.000
No	1 (0.3%)	0 (0.0%)	
Yes	330 (99.7%)	84 (100.0%)	
fliH			1.000
No	329 (99.4%)	84 (100.0%)	
Yes	2 (0.6%)	0 (0.0%)	
fliI			1.000
No	1 (0.3%)	0 (0.0%)	
Yes	330 (99.7%)	84 (100.0%)	
fliK			0.465
No	325 (98.2%)	84 (100.0%)	
Yes	6 (1.8%)	0 (0.0%)	
fliL			1.000
No	1 (0.3%)	0 (0.0%)	
Yes	330 (99.7%)	84 (100.0%)	
fliM			1.000
Yes	331 (100.0%)	84 (100.0%)	
fliN			1.000
Yes	331 (100.0%)	84 (100.0%)	
fliP			1.000
No	1 (0.3%)	0 (0.0%)	
Yes	330 (99.7%)	84 (100.0%)	
fliQ			1.000
Yes	331 (100.0%)	84 (100.0%)	
fliR			1.000
No	1 (0.3%)	0 (0.0%)	
Yes	330 (99.7%)	84 (100.0%)	
fliS			1.000
Yes	331 (100.0%)	84 (100.0%)	
fliW			1.000
Yes	331 (100.0%)	84 (100.0%)	

Campylobacter jejuni

Gene	Food (*n* =285) Count (%)	Human (*n* =422) Count (%)	Chi-square *p* -value^	
fliY			1.000	
Yes	285 (100.0%)	422 (100.0%)		
maf4			0.449	
No	261 (91.9%)	378 (89.8%)		
Yes	23 (8.1%)	43 (10.2%)		
motA			1.000	
Yes	285 (100.0%)	421 (100.0%)		
motB			1.000	
Yes	285 (100.0%)	422 (100.0%)		
pflA			1.000	
No	1 (0.4%)	2 (0.5%)		
Yes	284 (99.6%)	419 (99.5%)		
pseA			0.883	
No	12 (4.2%)	20 (4.7%)		
Yes	273 (95.8%)	402 (95.3%)		
pseB			1.000	
No	0 (0.0%)	1 (0.2%)		
Yes	285 (100.0%)	420 (99.8%)		
pseC			1.000	
Yes	285 (100.0%)	422 (100.0%)		
pseD/maf2			0.150	
No	221 (77.8%)	347 (84.0%)		
Yes	63 (22.2%)	66 (16.0%)		
pseE/maf5			0.009	*
No	81 (28.4%)	161 (38.2%)		
Yes	204 (71.6%)	261 (61.8%)		
pseF			1.000	
Yes	285 (100.0%)	422 (100.0%)		
pseG			0.843	
No	1 (0.4%)	0 (0.0%)		
Yes	284 (99.6%)	422 (100.0%)		
pseH			0.666	
No	12 (4.2%)	22 (5.2%)		
Yes	273 (95.8%)	400 (94.8%)		
pseI			1.000	
Yes	285 (100.0%)	422 (100.0%)		
ptmA			0.437	
No	57 (20.0%)	96 (22.7%)		
Yes	228 (80.0%)	326 (77.3%)		
ptmB			0.455	
No	45 (15.8%)	77 (18.2%)		
Yes	240 (84.2%)	345 (81.8%)		
rpoN			0.843	
No	1 (0.4%)	0 (0.0%)		
Yes	284 (99.6%)	421 (100.0%)		
jlpA			1.000	
No	1 (0.4%)	1 (0.2%)		
Yes	284 (99.6%)	421 (99.8%)		
Cj1135			0.787	
No	62 (21.8%)	87 (20.6%)		
Yes	223 (78.2%)	335 (79.4%)		
Cj1136			0.002	*
No	218 (76.5%)	276 (65.4%)		
Yes	67 (23.5%)	146 (34.6%)		

Campylobacter coli

Gene	Food (*n* =331) Count (%)	Human (*n* =84) Count (%)	Chi-square *p* -value^
fliY			1.000
No	1 (0.3%)	0 (0.0%)	
Yes	330 (99.7%)	84 (100.0%)	
maf4			0.167
No	315 (95.2%)	76 (90.5%)	
Yes	16 (4.8%)	8 (9.5%)	
motA			1.000
Yes	331 (100.0%)	84 (100.0%)	
motB			N/A
No	331 (100.0%)	84 (100.0%)	
pflA			1.000
No	2 (0.6%)	1 (1.2%)	
Yes	329 (99.4%)	83 (98.8%)	
pseA			0.821
No	12 (3.6%)	2 (2.4%)	
Yes	319 (96.4%)	82 (97.6%)	
pseB			1.000
No	1 (0.3%)	0 (0.0%)	
Yes	330 (99.7%)	84 (100.0%)	
pseC			1.000
Yes	331 (100.0%)	84 (100.0%)	
pseD/maf2			0.041
No	251 (75.8%)	73 (86.9%)	
Yes	80 (24.2%)	11 (13.1%)	
pseE/maf5			0.211
No	89 (26.9%)	29 (34.5%)	
Yes	242 (73.1%)	55 (65.5%)	
pseF			1.000
Yes	331 (100.0%)	84 (100.0%)	
pseG			0.787
No	8 (2.4%)	1 (1.2%)	
Yes	323 (97.6%)	83 (98.8%)	
pseH			0.865
No	28 (8.5%)	6 (7.1%)	
Yes	303 (91.5%)	78 (92.9%)	
pseI			1.000
Yes	331 (100.0%)	84 (100.0%)	
ptmA			1.000
No	82 (24.8%)	21 (25.0%)	
Yes	249 (75.2%)	63 (75.0%)	
ptmB			0.537
No	20 (6.0%)	3 (3.6%)	
Yes	311 (94.0%)	81 (96.4%)	
rpoN			1.000
No	1 (0.3%)	0 (0.0%)	
Yes	330 (99.7%)	84 (100.0%)	
jlpA			N/A
No	331 (100.0%)	84 (100.0%)	
Cj1135			
No	306 (92.4%)	77 (91.7%)	
Yes	25 (7.6%)	7 (8.3%)	
Cj1136			N/A
No	331 (100.0%)	84 (100.0%)	

	Campylobacter jejuni				***Campylobacter coli***		
	Food (*n* =285)	Human (*n* =422)	Chi-square		Food (*n* =331)	Human (*n* =84)	Chi-square
Gene	Count (%)	Count (%)	*p* -value^	Gene	Count (%)	Count (%)	*p* -value^
Cj1137c			0.982	***Cj1137c***			N/A
No	186 (65.3%)	277 (65.6%)		No	331 (100.0%)	84 (100.0%)	
Yes	99 (34.7%)	145 (34.4%)					
Cj1138			0.000 *	***Cj1138***			N/A
No	246 (86.3%)	276 (65.4%)		No	331 (100.0%)	84 (100.0%)	
Yes	39 (13.7%)	146 (34.6%)					
cstIII			0.857	***cstIII***			N/A
No	183 (64.2%)	275 (65.2%)		No	331 (100.0%)	84 (100.0%)	
Yes	102 (35.8%)	147 (34.8%)					
gmhA			1.000	***gmhA***			1.000
Yes	285 (100.0%)	419 (100.0%)		Yes	331 (100.0%)	84 (100.0%)	
gmhA2			1.000	***gmhA2***			0.250
No	74 (26.1%)	109 (25.8%)		No	216 (65.3%)	61 (72.6%)	
Yes	210 (73.9%)	313 (74.2%)		Yes	115 (34.7%)	23 (27.4%)	
gmhB			1.000	***gmhB***			1.000
No	0 (0.0%)	1 (0.2%)		Yes	331 (100.0%)	84 (100.0%)	
Yes	285 (100.0%)	421 (99.8%)					
hldD			1.000	***hldD***			1.000
No	0 (0.0%)	1 (0.2%)		Yes	331 (100.0%)	84 (100.0%)	
Yes	285 (100.0%)	421 (99.8%)					
hldE			1.000	***hldE***			1.000
Yes	285 (100.0%)	421 (100.0%)		No	1 (0.3%)	0 (0.0%)	
				Yes	330 (99.7%)	84 (100.0%)	
htrB			1.000	***htrB***			0.198
No	0 (0.0%)	1 (0.2%)		No	330 (99.7%)	82 (97.6%)	
Yes	285 (100.0%)	421 (99.8%)		Yes	1 (0.3%)	2 (2.4%)	
neuA			0.908	***neuA***			N/A
No	183 (64.2%)	274 (64.9%)		No	331 (100.0%)	84 (100.0%)	
Yes	102 (35.8%)	148 (35.1%)					
neuB			0.833	***neuB***			0.581
No	182 (63.9%)	274 (64.9%)		No	321 (97.0%)	83 (98.8%)	
Yes	103 (36.1%)	148 (35.1%)		Yes	10 (3.0%)	1 (1.2%)	
neuC			0.790	***neuC***			0.581
No	190 (66.7%)	276 (65.4%)		No	321 (97.0%)	83 (98.8%)	
Yes	95 (33.3%)	146 (34.6%)		Yes	10 (3.0%)	1 (1.2%)	
waaC			1.000	***waaC***			0.434
Yes	285 (100.0%)	422 (100.0%)		No	5 (1.5%)	3 (3.6%)	
				Yes	326 (98.5%)	81 (96.4%)	
waaF			1.000	***waaF***			0.585
Yes	285 (100.0%)	422 (100.0%)		No	3 (0.9%)	2 (2.4%)	
				Yes	328 (99.1%)	82 (97.6%)	
waaV			0.006 *	***waaV***			1.000
No	10 (3.5%)	2 (0.5%)		No	1 (0.3%)	0 (0.0%)	
Yes	275 (96.5%)	420 (99.5%)		Yes	330 (99.7%)	84 (100.0%)	
wlaN			0.014 *	***wlaN***			N/A
No	217 (76.1%)	284 (67.3%)		No	331 (100.0%)	84 (100.0%)	
Yes	68 (23.9%)	138 (32.7%)					
porA			1.000	***porA***			1.000
Yes	285 (100.0%)	422 (100.0%)		Yes	331 (100.0%)	84 (100.0%)	
pebA			1.000	***pebA***			1.000
Yes	285 (100.0%)	422 (100.0%)		No	1 (0.3%)	0 (0.0%)	
				Yes	330 (99.7%)	84 (100.0%)	
virB10			N/A	***virB10***			N/A
No	278 (97.5%)	0		No	326 (98.5%)	0	
Yes	7 (2.5%)	0		Yes	5 (1.5%)	0	

	Campylobacter jejuni				*Campylobacter coli*		
Gene	Food (*n* =285) Count (%)	Human (*n* =422) Count (%)	Chi-square *p* -value^	Gene	Food (*n* =331) Count (%)	Human (*n* =84) Count (%)	Chi-square *p* -value^
virB4			N/A	***virB4***			N/A
No	278 (97.5%)	0		No	326 (98.5%)	0	
Yes	7 (2.5%)	0		Yes	5 (1.5%)	0	
virB8			N/A	***virB8***			N/A
No	278 (97.5%)	0		No	326 (98.5%)	0	
Yes	7 (2.5%)	0		Yes	5 (1.5%)	0	
virB9			N/A	***virB9***			N/A
No	278 (97.5%)	0		No	326 (98.5%)	0	
Yes	7 (2.5%)	0		Yes	5 (1.5%)	0	
virD4			N/A	***virD4***			N/A
No	278 (97.5%)	0		No	326 (98.5%)	0	
Yes	7 (2.5%)	0		Yes	5 (1.5%)	0	

S1 File. Random Forest model outputs determining virulence genes that predict hospitalisation or length of diarrhoeal illness in Australia, 2018–2019.

Notes for interpretation: mtry: number of variables randomly sampled as candidates at each split. lambda.freqs: tree depth regularisation parameter.

C. jejuni

Method	Number of isolates	Number of virulence genes	Outcome	Number of trees/splits
Random forest	422	58	Hospitalisation	100

```
  mtry  Accuracy  Kappa
   2    0.7610     0.00000000
   8    0.7510    -0.01166667
  14    0.7340    -0.01166667
  20    0.7245    -0.01446970
  26    0.7040    -0.02946970
  33    0.7020    -0.02696970
  39    0.7025    -0.01946970
  45    0.7025    -0.01946970
  51    0.7000    -0.02280303
  58    0.7025    -0.02113636

Kappa was used to select the optimal model using the largest value.
The final value used for the model was mtry = 2.
```

Method	Number of isolates	Number of virulence genes	Outcome	Number of trees/splits
Binary discriminant analysis	422	58	Hospitalisation	100

```
  lambda.freqs  Accuracy  Kappa
  0.0           0.6120    0.06726522
  0.5           0.5885    0.03644054
  1.0           0.5755    0.03744054

Kappa was used to select the optimal model using the largest value.
The final value used for the model was lambda.freqs = 0.
```

Method	Number of isolates	Number of virulence genes	Outcome	Number of trees/splits
Logic regression	422	58	Hospitalisation	100

```
  ntrees  treesize  Accuracy  Kappa
  2        4        0.7515    -0.01250000
  2        8        0.7485    -0.01666667
  2       16        0.7255    -0.01392857
  3        4        0.7545     0.00500000
  3        8        0.7325    -0.02280303
  3       16        0.7280    -0.03000000
  4        4        0.7345     0.01450216
  4        8        0.7310    -0.01761905
  4       16        0.7245    -0.01916667

Kappa was used to select the optimal model using the largest value.
The final values used for the model were treesize = 4 and ntrees = 4.
```

Method	Number of isolates	Number of virulence genes	Outcome	Number of trees/splits
Random forest	420	58	Length of illness	100

```
  mtry  Accuracy  Kappa
   2    0.5505     0.07782051
   8    0.4965    -0.01285315
  14    0.4660    -0.07527739
  20    0.4610    -0.08265501
  26    0.4825    -0.03990443
  33    0.4785    -0.04564868
  39    0.4900    -0.02548718
  45    0.4820    -0.04185082
  51    0.4915    -0.02230536
  58    0.4860    -0.03100833

Kappa was used to select the optimal model using the largest value.
The final value used for the model was mtry = 2.
```

Method	Number of isolates	Number of virulence genes	Outcome	Number of trees/splits
Binary discriminant analysis	420	58	Length of illness	100

```
  lambda.freqs  Accuracy   Kappa
  0.0           0.5156667  0.022220113
  0.5           0.5101667  0.012523144
  1.0           0.5028333  0.002987013

Kappa was used to select the optimal model using the largest value.
The final value used for the model was lambda.freqs = 0.
```

Method	Number of isolates	Number of virulence genes	Outcome	Number of trees/splits
Logic regression	420	58	Length of illness	100

```
  ntrees  treesize  Accuracy   Kappa
  2        4        0.5181667   0.0001298701
  2        8        0.5095000   0.0038431568
  2       16        0.5076667  -0.0104745255
  3        4        0.5106667  -0.0109324009
  3        8        0.4685000  -0.0823672994
  3       16        0.4856667  -0.0481518482
  4        4        0.5156667   0.0106909757
  4        8        0.4970000  -0.0270812521
  4       16        0.4891667  -0.0511948052

Kappa was used to select the optimal model using the largest value.
The final values used for the model were treesize = 4 and ntrees = 4.
```

C. coli

Method	Number of isolates	Number of virulence genes	Outcome	Number of trees/splits
Random forest	84	43	Hospitalisation	10

```
  mtry  Accuracy   Kappa
   2    0.8246032   0.00000000
   6    0.8246032   0.00000000
  11    0.7898810  -0.04906832
  15    0.7599206  -0.03377786
  20    0.7724206   0.03019730
  24    0.7599206   0.01591158
  29    0.7724206   0.03019730
  33    0.7724206   0.03019730
  38    0.7724206   0.03019730
  43    0.7724206   0.03019730

Kappa was used to select the optimal model using the largest value.
The final value used for the model was mtry = 20.
```

Method	Number of isolates	Number of virulence genes	Outcome	Number of trees/splits
Binary discriminant analysis	84	43	Hospitalisation	10

```
  lambda.freqs  Accuracy   Kappa
  0.0           0.7884921  -0.002484472
  0.5           0.6890873  -0.004884144
  1.0           0.6432540  -0.003069763

Kappa was used to select the optimal model using the largest value.
The final value used for the model was lambda.freqs = 0.
```

Method	Number of isolates	Number of virulence genes	Outcome	Number of trees/splits
Logic regression	84	43	Hospitalisation	10

```
  ntrees  treesize  Accuracy   Kappa
  2        4        0.7996032   0.118016076
  2        8        0.7373016  -0.050854037
  2       16        0.7563492   0.023728387
  3        4        0.7123016   0.018635169
  3        8        0.7581349   0.152808547
  3       16        0.7613095   0.008868185
  4        4        0.7025794  -0.037576011
  4        8        0.7674603   0.136833977
  4       16        0.7724206   0.086259489

Kappa was used to select the optimal model using the largest value.
The final values used for the model were treesize = 8 and ntrees = 3.
```

Method	Number of isolates	Number of virulence genes	Outcome	Number of trees/splits
Random forest	83	43	Length of illness	10

```
  mtry  Accuracy   Kappa
   2    0.5321429  -0.04210526
   6    0.4817460  -0.07620545
  11    0.4359127  -0.16782489
  15    0.4470238  -0.14717711
  20    0.4359127  -0.16894098
  24    0.4011905  -0.22447886
  29    0.3998016  -0.20859735
  33    0.3998016  -0.20971344
  38    0.3886905  -0.23036122
  43    0.3748016  -0.25991314

Kappa was used to select the optimal model using the largest value.
The final value used for the model was mtry = 2.
```

Method	Number of isolates	Number of virulence genes	Outcome	Number of trees/splits
Binary discriminant analysis	83	43	Length of illness	10

```
  lambda.freqs  Accuracy   Kappa
  0.0           0.4277778  -0.12429603
  0.5           0.4549603  -0.04756695
  1.0           0.4295635  -0.08230281

Kappa was used to select the optimal model using the largest value.
The final value used for the model was lambda.freqs = 0.5.
```

Method	Number of isolates	Number of virulence genes	Outcome	Number of trees/splits
Logic regression	83	43	Length of illness	10

```
  ntrees  treesize  Accuracy   Kappa
  2        4        0.4361111  -0.14684789
  2        8        0.5222222  -0.02134690
  2       16        0.5111111  -0.03370690
  3        4        0.5208333  -0.02563855
  3        8        0.4958333  -0.09074844
  3       16        0.5097222  -0.04834227
  4        4        0.4472222  -0.17425502
  4        8        0.4583333  -0.12886430
  4       16        0.4111111  -0.22535465

Kappa was used to select the optimal model using the largest value.
The final values used for the model were treesize = 8 and ntrees = 2.
```

S2 File: Random Forest model outputs determining virulence genes that predict a human case compared with retail meat and offal isolates in Australia, 2018–2019.

Notes for interpretation: mtry: number of variables randomly sampled as candidates at each split. lambda.freqs: tree depth regularisation parameter.

C. jejuni

Method	Number of isolates	Number of virulence genes	Outcome	Number of trees/splits
Random forest	707	65	Case illness	100

```
  mtry  Accuracy   Kappa
   2    0.5989881  0.005714286
   9    0.7405357  0.437036730
  16    0.7551190  0.482911542
  23    0.7543452  0.485038427
  30    0.7625000  0.503695179
  37    0.7648214  0.508544008
  44    0.7635714  0.503578584
  51    0.7633929  0.504536306
  58    0.7566071  0.490144588
  65    0.7563690  0.487264723

Kappa was used to select the optimal model using the largest value.
The final value used for the model was mtry = 37.
```

See S1 Figure for relative importance of specified virulence genes.

Method	Number of isolates	Number of virulence genes	Outcome	Number of trees/splits
Binary discriminant analysis	707	65	Case illness	100

```
  lambda.freqs  Accuracy   Kappa
  0.0           0.6035119  0.1741997
  0.5           0.5927976  0.1692719
  1.0           0.5854762  0.1772702

Kappa was used to select the optimal model using the largest value.
The final value used for the model was lambda.freqs = 1.
```

Method	Number of isolates	Number of virulence genes	Outcome	Number of trees/splits
Logic regression	707	65	Case illness	100

```
  ntrees  treesize  Accuracy   Kappa
  2        4        0.7486310  0.4118253
  2        8        0.7380357  0.3911470
  2       16        0.7488690  0.4179584
  3        4        0.7395833  0.3915202
  3        8        0.7429167  0.3995143
  3       16        0.7517262  0.4267998
  4        4        0.7408333  0.3973660
  4        8        0.7468452  0.4126220
  4       16        0.7251786  0.3751444

Kappa was used to select the optimal model using the largest value.
The final values used for the model were treesize = 16 and ntrees = 3.
```

C. coli

Method	Number of isolates	Number of virulence genes	Outcome	Number of trees/splits
Random forest	415	72	Case illness	100

```
  mtry  Accuracy  Kappa
   2    0.8035     0.000000000
   9    0.7985    -0.007936508
  17    0.8005     0.011904762
  25    0.7960     0.012896825
  33    0.7960     0.012896825
  40    0.7915     0.009803922
  48    0.7940     0.021568627
  56    0.7915     0.017647059
  64    0.7940     0.021568627
  72    0.7915     0.017647059

Kappa was used to select the optimal model using the largest value.
The final value used for the model was mtry = 48.
```

Method	Number of isolates	Number of virulence genes	Outcome	Number of trees/splits
Binary discriminant analysis	415	72	Case illness	100

```
  lambda.freqs  Accuracy   Kappa
  0.0           0.7873333  0.002906977
  0.5           0.6475000  0.015638528
  1.0           0.5188333  0.086611138

Kappa was used to select the optimal model using the largest value.
The final value used for the model was lambda.freqs = 1.
```

Method	Number of isolates	Number of virulence genes	Outcome	Number of trees/splits
Logic regression	415	72	Case illness	100

```
  ntrees  treesize  Accuracy   Kappa
  2        4        0.7965000  -0.010912698
  2        8        0.7990000  -0.006944444
  2       16        0.7880000  -0.013257576
  3        4        0.7855000  -0.027777778
  3        8        0.7741667  -0.036274510
  3       16        0.7950000   0.031385281
  4        4        0.7855000  -0.018217893
  4        8        0.7855000  -0.018849206
  4       16        0.7865000   0.038235294

Kappa was used to select the optimal model using the largest value.
The final values used for the model were treesize = 16 and ntrees = 4.
```

S1 Fig. Prevalence of virulence genes and gene clustering by multi-locus sequence type (MLST) for C. jejuni human isolates. *The colour scale represents the proportion of isolates within each MLST expressing each virulence gene.*

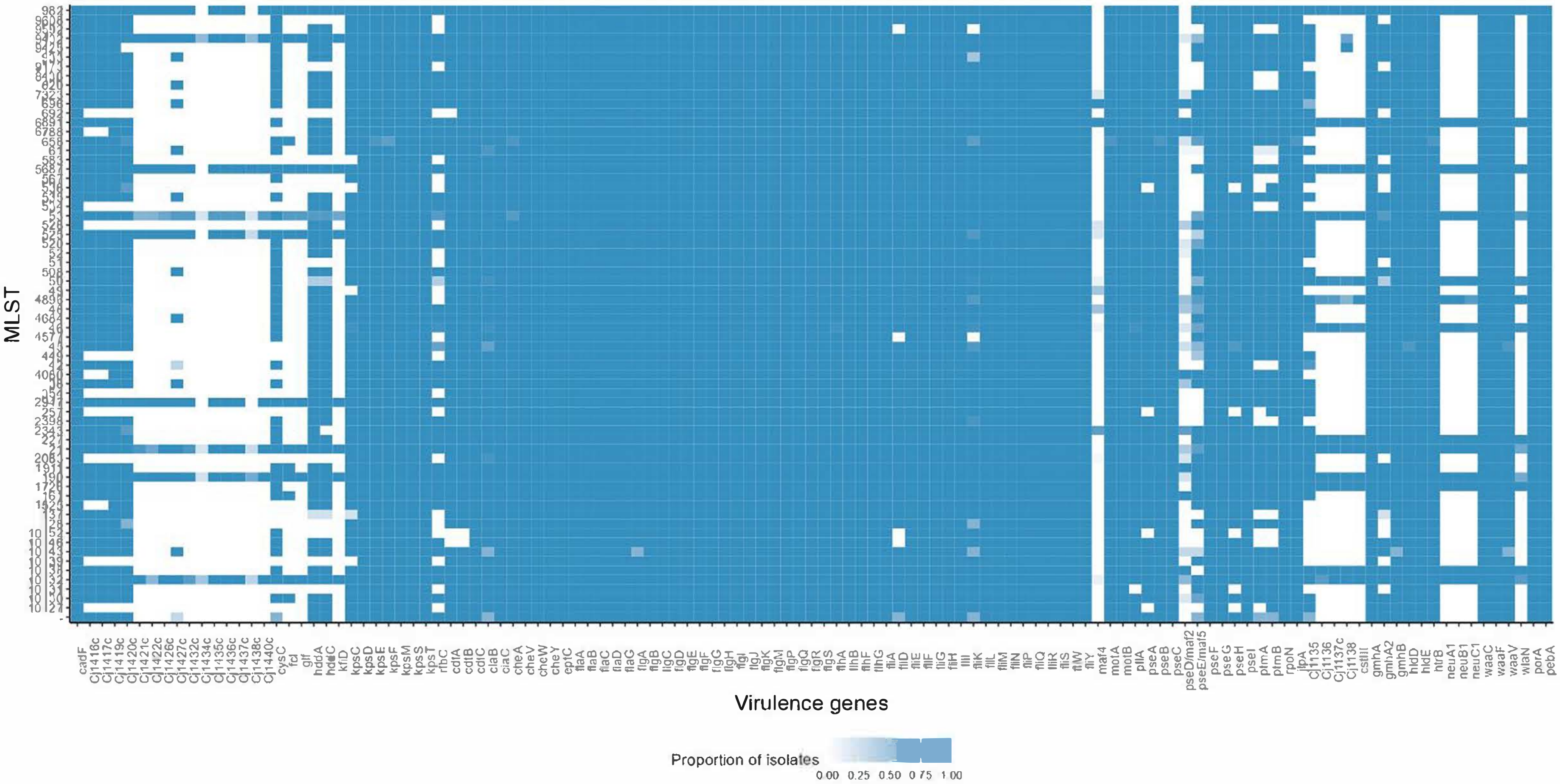

S2 Fig. Prevalence of virulence genes and gene clustering by multi-locus sequence type (MLST) for C. coli human isolates. *The colour scale represents the proportion of isolates within each MLST expressing each virulence gene.*

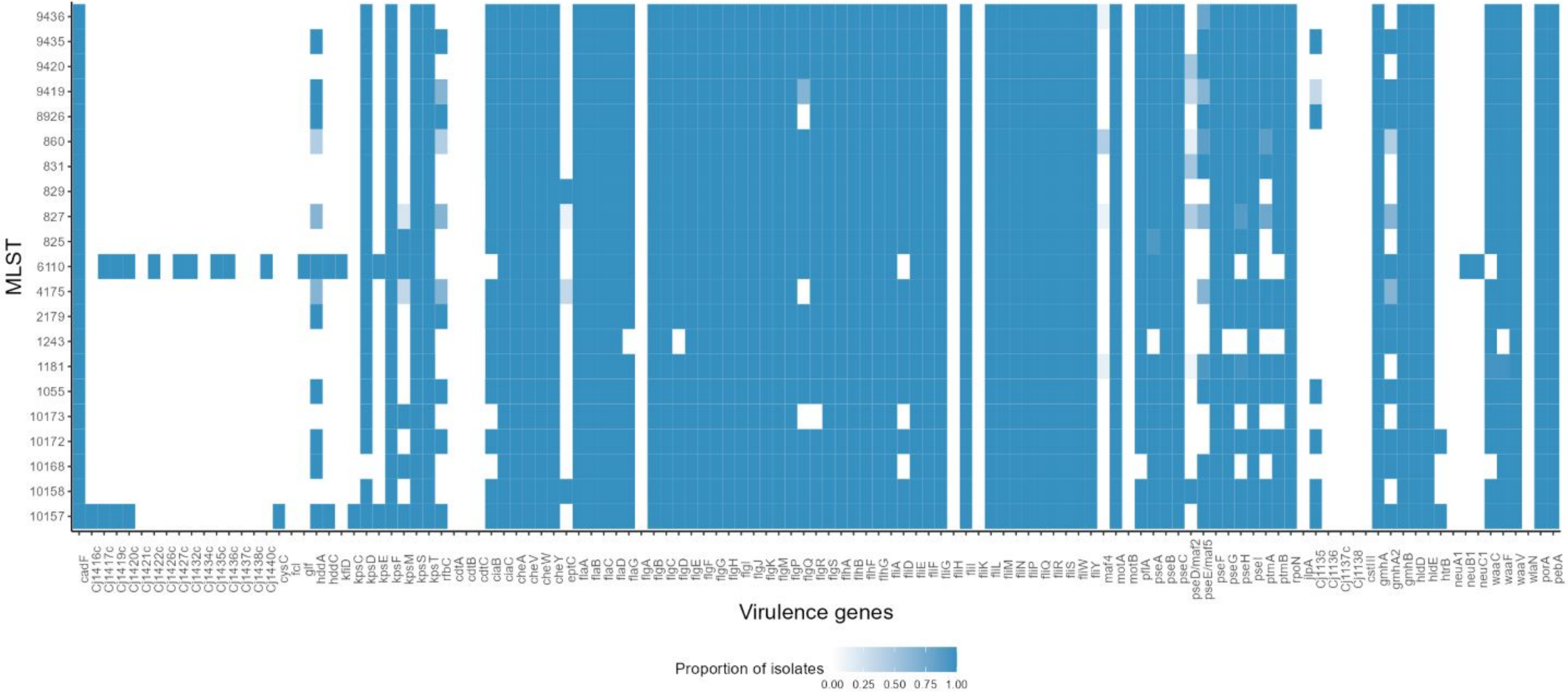

S3 Fig. Maximum likelihood phylogenetic tree showing the core genome relationship between C. jejuni isolates (n = 422) from humans in the Australian Capital Territory (ACT), New South Wales (NSW), and Queensland (Qld). *The circle lanes from inner to outer indicate jurisdiction, multilocus sequence type (MLST), the number of class of antimicrobial genotype detected, and the number and trait class of virulence genotype detected.*

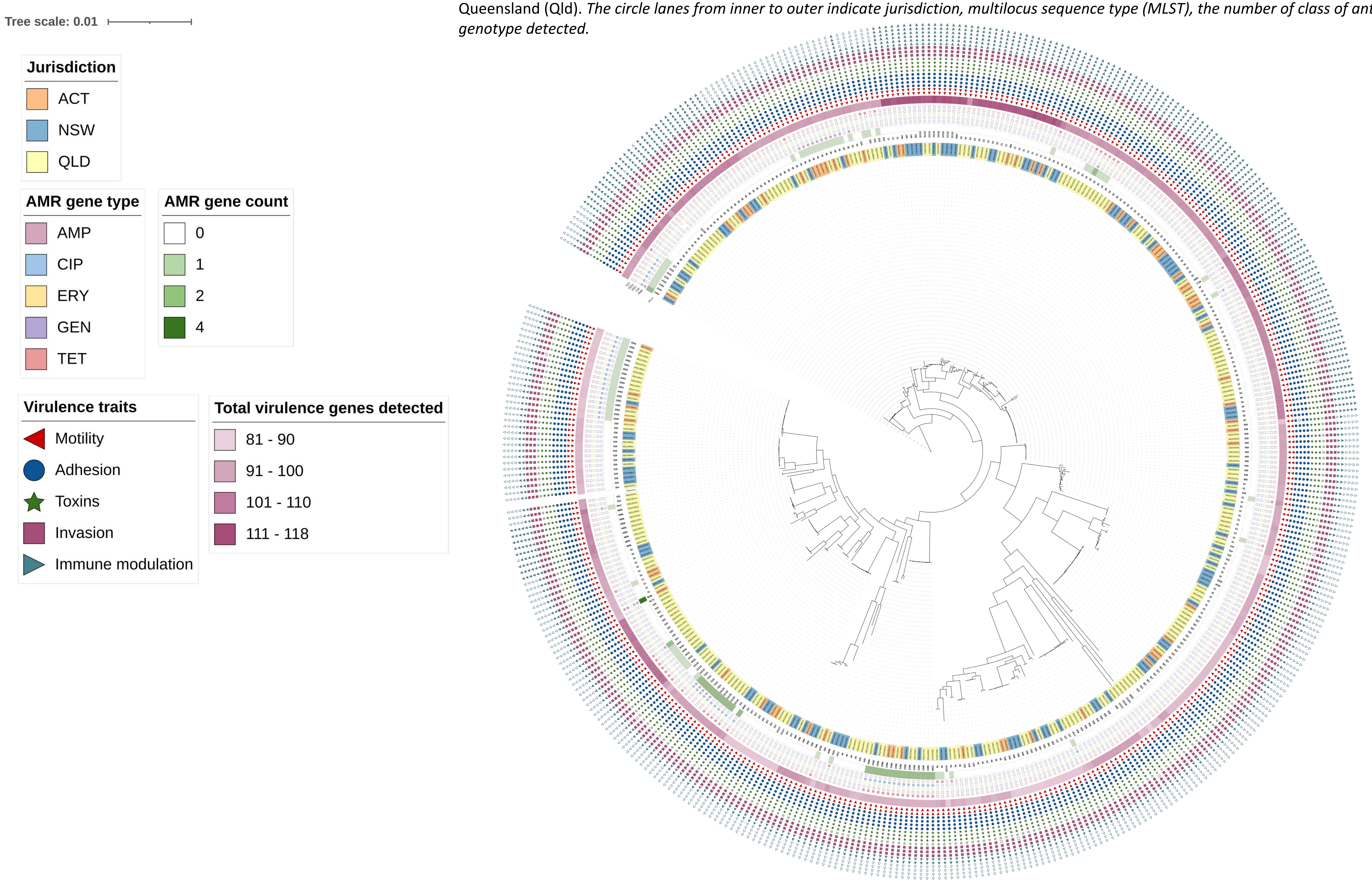

S4 Fig. Maximum likelihood phylogenetic tree showing the core genome relationship between C. coli isolates (n = 84) from humans in the Australian Capital Territory (ACT), New South Wales (NSW), and Queensland (QLD). *The circle lanes from inner to outer indicate jurisdiction, multilocus sequence type (MLST), the number and class of antimicrobial genotype detected, and the number and trait class of virulence genotype detected.*

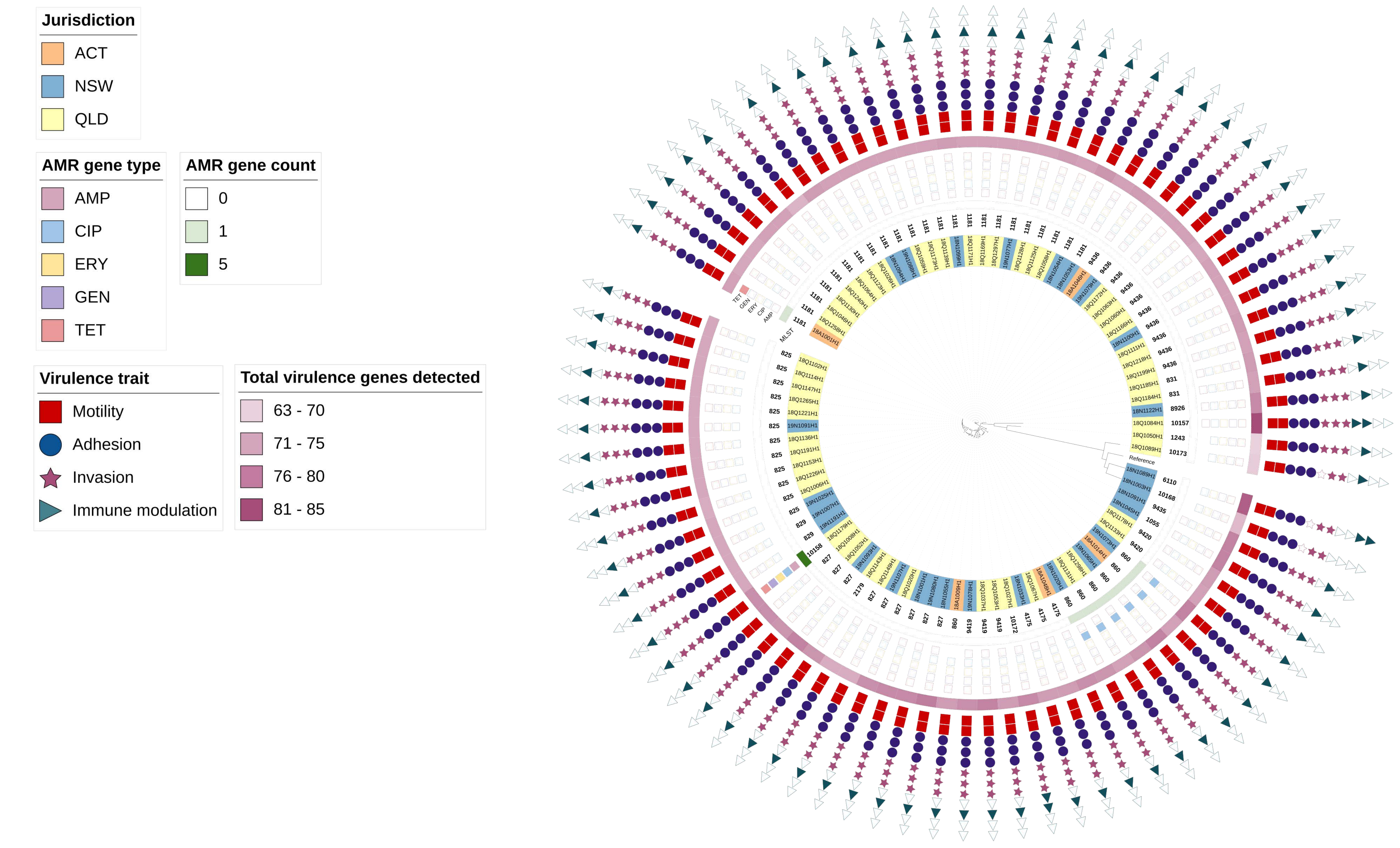

S5 Fig. Relative importance by Gini* coefficient of *Campylobacter jejuni* virulence genes for predicting case isolates^ compared with meat isolates in Australia, 2018–2019. ** Mean decrease in Gini coefficient measures how much of each variable contributes to the homogeneity of the nodes and leaves in the random forest. The higher the value of mean decrease Gini score, the higher the importance of the variable. Values should be considered relative to those of other variables rather than absolute values. ^Genes more common in case isolates include* fliK, Cj1136, Cj1138, Cj1135, maf4, neuC, rfbC, wlaN, cysC, Cj1422c, Cj1421c, gmhA2, kpsC, waaV, fcl, Cj1420c, ciaB*, and* Cj1419c *although not all differences in gene prevalence are significant (p <0.05).*

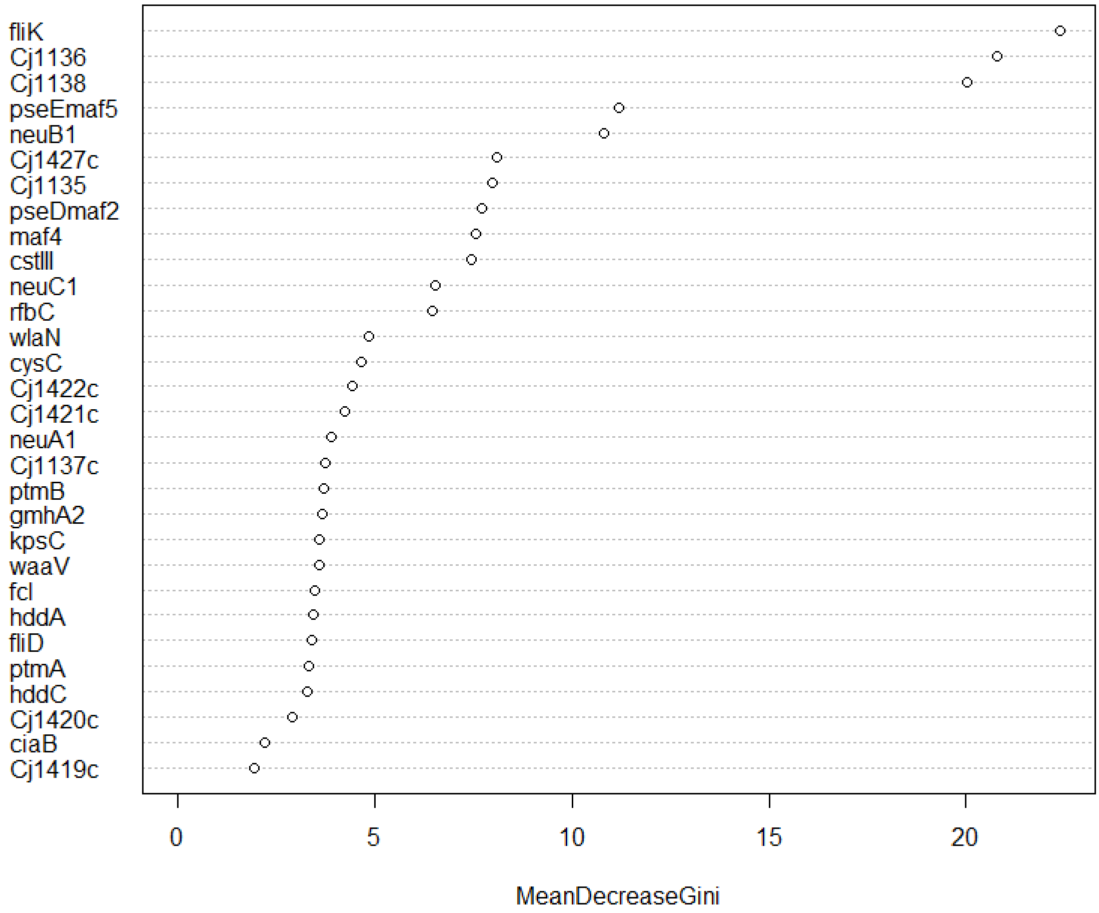

Supplement: Supplementary material 1 [file mgen-10-1174-s001.pdf]
